# Total evidence time-scaled phylogenetic and biogeographic models for the evolution of sea cows (Sirenia, Afrotheria)

**DOI:** 10.7717/peerj.13886

**Published:** 2022-08-25

**Authors:** Steven Heritage, Erik R. Seiffert

**Affiliations:** 1 Duke Lemur Center Museum of Natural History, Duke University, Durham, NC, USA; 2 Department of Integrative Anatomical Sciences, Keck School of Medicine of USC, University of Southern California, Los Angeles, CA, USA; 3 Department of Mammalogy, Natural History Museum of Los Angeles County, Los Angeles, CA, USA

**Keywords:** Sirenia, Sea Cows, Manatees, Dugongs, Phylogenetics, Bayesian Tip-Dating, Historical Biogeography, Afrotheria, Marine Mammals, Paleontology

## Abstract

Molecular phylogenetic studies that have included sirenians from the genera *Trichechus*, *Dugong*, and *Hydrodamalis* have resolved their interrelationships but have yielded divergence age estimates that are problematically discordant. The ages of these lineage splits have profound implications for how to interpret the sirenian fossil record—including clade membership, biogeographic patterns, and correlations with Earth history events. In an effort to address these issues, here we present a total evidence phylogenetic analysis of Sirenia that includes living and fossil species and applies Bayesian tip-dating methods to estimate their interrelationships and divergence times. In addition to extant sirenians, our dataset includes 56 fossil species from 106 dated localities and numerous afrotherian outgroup taxa. Genetic, morphological, temporal, and biogeographic data are assessed simultaneously to bring all available evidence to bear on sirenian phylogeny. The resulting time-tree is then used for Bayesian geocoordinates reconstruction analysis, which models ancestral geographic areas at splits throughout the phylogeny, thereby allowing us to infer the direction and timing of dispersals. Our results suggest that Pan-Sirenia arose in North Africa during the latest Paleocene and that the Eocene evolution of stem sirenians was primarily situated in the Tethyan realm. In the late Eocene, some lineages moved into more northern European latitudes, an area that became the source region for a key trans-Atlantic dispersal towards the Caribbean and northern-adjacent west Atlantic. This event led to the phylogenetic and biogeographic founding of crown Sirenia with the Dugongidae-Trichechidae split occurring at the Eocene-Oligocene boundary (~33.9 Ma), temporally coincident with the onset of dropping global sea levels and temperatures. This region became the nexus of sirenian diversification and supported taxonomically-rich dugongid communities until the earliest Pliocene. The Dugonginae-Hydrodamalinae split occurred near Florida during the early Miocene (~21.2 Ma) and was followed by a west-bound dispersal that gave rise to the Pacific hydrodamalines. The late middle Miocene (~12.2 Ma) split of *Dugong* from all other dugongines also occurred near Florida and our analyses suggest that the Indo-Pacific distribution of modern dugongs is the result of a trans-Pacific dispersal. From at least the early Miocene, trichechid evolution was based entirely in South America, presumably within the Pebas Wetlands System. We infer that the eventual establishment of Amazon drainage into the South Atlantic allowed the dispersal of *Trichechus* out of South America no earlier than the mid-Pliocene. Our analyses provide a new temporal and biogeographic framework for understanding major events in sirenian evolution and their possible relationships to oceanographic and climatic changes. These hypotheses can be further tested with the recovery and integration of new fossil evidence.

## Introduction

The mammalian order Sirenia, commonly called the sea cows, includes only four living species—the dugong (genus *Dugong*, family Dugongidae) and three manatees (genus *Trichechus*, family Trichechidae). Sirenians are unique in being the only fully aquatic mammals that are also obligate herbivores, and their habitats are predominantly warm, near-shore, and relatively shallow waters that accommodate the vegetative growth that comprises their diets (Marsh, 2014; O’Shea, 2014). The fossil record of sea cows begins near the onset of the middle Eocene, about 48 million years ago, and since that time their evolving diversity and biogeography has ensued to leave fossil occurrences on every continent except Antarctica (Marsh, O’Shea & Reynolds, 2012). While the extant species of sea cows lack hindlimbs and are equipped with several anatomical adaptations that suit them to wholly aquatic lifeways, some of the earliest fossil sirenians were, by contrast, quadrupedal and amphibious (Domning, 2001a; Domning, 2009).

**Figure 1:**
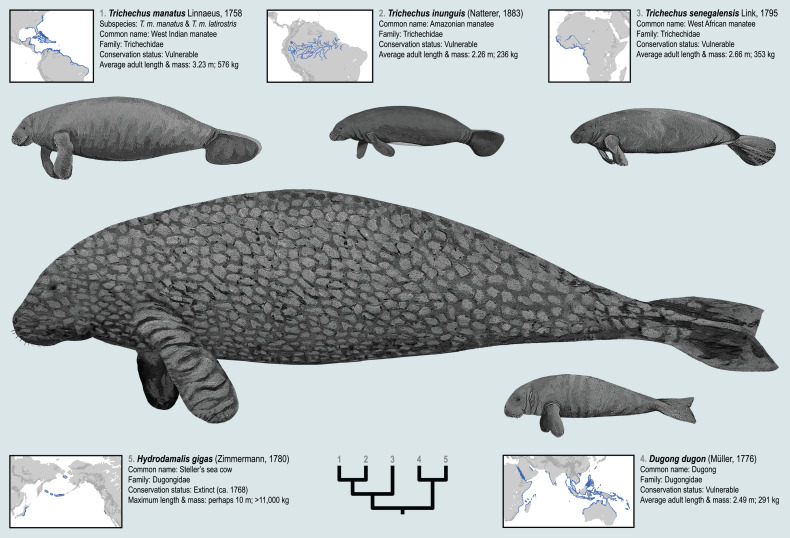
The four extant sirenian species and Steller’s sea cow (recently extinct). All five taxa are represented by DNA sequences. Cladogram of molecular phylogenetic relationships is a composite from Springer et al. (2015) and de Souza et al. (2021). Species distribution maps are adapted from the IUCN (2021) Red List of Threatened Species and from empirical *H. gigas* occurrence localities included in this study. Body size estimates are from multiple sources discussed in the *OSF Data Supplement*. Figured body sizes are at relative scale. Original paintings by Christy Holton.

The geographic distributions of the living sirenian species are mapped in Fig. 1. The West Indian manatee (*Trichechus manatus*) occurs throughout the Caribbean, in parts of the Gulf of Mexico, and along the Atlantic coasts of North and South America—while the Amazonian manatee (*Trichechus inunguis*) inhabits the river systems of the Amazon Basin. The West African manatee (*Trichechus senegalensis*) occurs along the Atlantic coasts of West and Central Africa and in several lake and river systems of that region which ultimately drain into the east Atlantic and Gulf of Guinea (O’Shea, 2014). Compared to the other living sea cow species, the dugong (*Dugong dugon*) has the broadest distribution—inhabiting tropical and subtropical waters of the Eastern Hemisphere from the coasts of East Africa and Madagascar to the Red Sea and Persian Gulf, along the western coast of India, and throughout the near-shore waters of Southeast Asia and Australasia all the way to New Caledonia (Marsh, 2014; Plön et al., 2019). The Recent species Steller’s sea cow (*Hydrodamalis gigas*, family Dugongidae; Fig. 1) was last reported alive in the middle of the 18^th^ century but has since been hunted to extinction (Turvey & Risley, 2006). Steller’s sea cow was much larger than the living sirenian species, was apparently adapted to colder waters, and is documented by Pacific Rim occurrences from Japan, to the Aleutian Islands, to Northern California (Domning, 1978; Furusawa, 2004). Substantial genomic data derived from relatively young specimens (<300 years old) have enabled robust phylogenetic placement of Steller’s sea cow among the extant sirenian genera (Springer et al., 2015). These five species, living and Recent, are only a narrow temporal sample from the order’s long history. Therefore, the evolutionary events that have led to this small group’s relatively widespread geographic range might be better understood through a deep-time lens and in a richer taxonomic context (see below).

Concerning their systematic position, sirenians unequivocally belong to the endemic Afro-Arabian clades Afrotheria and Paenungulata (Murphy et al., 2001; Springer et al., 2003; Meredith et al., 2011), and based on recent large-scale genetic datasets, Sirenia is probably the extant sister taxon of the order Proboscidea (elephants) to the exclusion of Hyracoidea (hyraxes or dassies) (*e.g*., Heritage, Seiffert & Borths, 2021; Poulakakis & Stamatakis, 2010; Schull et al., 2022; Springer et al., 2015). Given that the oldest paenungulate fossils are from the Paleocene of Afro-Arabia (*e.g*., Gheerbrant, Bouya & Amaghzaz, 2012), it seems likely that the sirenian stem lineage arose on the same landmass during the very early Cenozoic. However, a temporal comparison of the earliest known sirenian fossils to the supposed age of the Sirenia-Proboscidea lineage split (*e.g*., ~59.2 Ma; Heritage, Seiffert & Borths, 2021) suggests that the first 10 million years (or so) of the order’s evolutionary history has not yet been documented by fossils. Conversely, the middle Eocene to Recent fossil record of sea cows is quite good, and provides evidence of times with much higher taxonomic and morphological diversity compared to the extant group—and notably so during the Oligo-Miocene interval (Domning, 2009). Sirenian fossils of Eocene age have evidenced several transitions in their highly derived anatomy including the loss of hindlimbs, modification of the forelimbs into “flippers”, alterations of the vertebral column and dental formula, and the development of pachyosteosclerotic ribs (Domning, 2001a; Springer et al., 2015; Vélez-Juarbe & Wood, 2018).

Most well-documented extinct sea cow species have already been scored for morphological characters, and these data have been employed several times to estimate relationships among living and fossil taxa using parsimony methods (*e.g*., Domning, 1994; Springer et al., 2015; Vélez-Juarbe & Wood, 2018). Abundant DNA evidence is also now available for all extant sirenian species (plus Steller’s sea cow), but molecular and morphological data have never been assessed simultaneously using statistical phylogenetic methods. Perhaps this is partly attributable to the two data types independently and strongly informing a *Dugong-Hydrodamalis* clade that is sister to *Trichechus* (Fig. 1).

Although the pattern of descent among living manatees and dugongs (plus Steller’s sea cow) can now be considered well-resolved, molecular-based models that have estimated the timing of lineage divergences have yielded wildly different ages for the Dugongidae-Trichechidae split (*i.e*., crown Sirenia) and the *Dugong-Hydrodamalis* split (*i.e*., crown Dugongidae *sensu* this study; see Results). Estimates for the age of crown Sirenia range from middle Eocene (~46.8 Ma, de Souza et al., 2021; ~41.5 Ma, Springer et al., 2015; ~41 Ma, Plön et al., 2019), to Oligocene (~31.4 Ma, Meredith et al., 2011; ~30.9 Ma, Heritage, Seiffert & Borths, 2021), to Miocene (~16.6 Ma, Poulakakis & Stamatakis, 2010; ~13 Ma, Upham, Esselstyn & Jetz, 2019; ~5.74 Ma, Phillips, 2016), while estimates for the *Dugong-Hydrodamalis* split range from Oligocene (~29 Ma, Plön et al., 2019; ~28.6 Ma, Springer et al., 2015) to Miocene (~15.1 Ma, Heritage, Seiffert & Borths, 2021). These disparate evolutionary timescales have dramatically different implications for interpretations of the sirenian fossil record, including clade membership and biogeography. Severe discrepancies in age estimates among these molecular studies are presumably due, in large part, to different fossil calibration strategies (*e.g*., which fossil taxa are selected, variable assumptions about the nested phylogenetic positions of fossil taxa, the number of fossil calibrations included in the analysis, etc.). For instance, Upham, Esselstyn & Jetz (2019) used only one fossil calibration within Afrotheria, whereas Heritage, Seiffert & Borths (2021) applied 15.

Rigorous, quantitative investigations of macroevolutionary phenomena, such as long-term anatomical transformations or shifts in geographic distribution, and the potential relationship of such phenomena to external drivers (*e.g*., geologic, oceanographic, climatic, and biotic events) can only be undertaken within the context of time-scaled phylogenetic hypotheses. Relatively recent advances in Bayesian phylogenetics (*i.e*., the development of total evidence Bayesian tip-dating methods) allow phylogeny and divergence times of living and fossil taxa to be estimated simultaneously from mixed data types (Ronquist et al., 2012a). These analyses use statistical models for both topological placements and relaxed clock branch lengths, thereby obviating the need for “expert” identification of appropriate fossil calibrations (Pozzi & Penna, 2022). In other words, the likelihood-evaluated phylogenetic placement of a dated fossil taxon within a tree automatically constrains a minimum age for its preceding tree node. Such analyses yield phylogenies with objective divergence age estimates which, in turn, are the critical frameworks against which to assess the pattern and rates of evolving character states and lineage diversity through time—among many other potential inferences.

In this contribution we combine existing morphological, molecular, temporal, and geographic data, and apply Bayesian tip-dating (BTD) phylogenetic methods, to bring all available evidence to bear on the evolutionary relationships of living and fossil sea cows. We then use this novel time-scaled tree—along with a large geospatial dataset containing geographic coordinates of fossil occurrences and extant species ranges—to estimate the ancestral geographic areas that correspond to tree nodes throughout sirenian phylogeny (*i.e*., Bayesian continuous-variable geographic reconstruction analysis) (Meade & Pagel, 2022). Our aim is to provide data-driven and algorithm-driven hypotheses for the relationships, divergence times, and ancestral biogeography of Sirenia that are explicitly testable, and which can be refined with the addition of new evidence. While previously published estimates of sirenian phylogeny have been derived from independent analyses of morphology or molecules, by incorporating multiple lines of evidence in a simultaneous assessment, our approach improves the phylogenetic explanatory power of the component data types (Nylander et al., 2004; Wortley & Scotland, 2006).

## Methods

### Data deposition

All data, analysis settings, and additional supplementary files necessary to replicate this study have been permanently archived at the Open Science Framework (OSF) repository (Foster & Deardorff, 2017). In-text references to this archive are: *OSF Data Supplement*. The supplement can be accessed at: https://doi.org/10.17605/OSF.IO/H7ZFD.

### DNA dataset

The molecular dataset compiled for this study includes DNA sequences from 50 taxa and samples all major groups within the afrotherian clade. Accession numbers for sequences retrieved from GenBank are reported in the *OSF Data Supplement*. Included in this sample are all four extant sirenian species (*Trichechus inunguis*, *Trichechus manatus*, *Trichechus senegalensis*, and *Dugong dugon*) and the recently extinct Steller’s sea cow (*Hydrodamalis gigas*). Also included are three recently extinct proboscideans (*Mammut americanum*, *Mammuthus primigenius*, and *Palaeoloxodon antiquus*). Data for the latter two proboscideans were sourced from genomic sequence read archives. The dataset comprises 32 gene segments (19 nuclear, 13 mitochondrial), all of which are amino-acid coding. Orthologous segments were aligned using the Geneious v7.1.7 (Kearse et al., 2012) translation-guided alignment tool and each alignment was manually trimmed to begin on codon position-1 and end on position-3. The concatenated supermatrix of these alignments amounts to 33,468 base positions (22,398 nuclear, 11,070 mitochondrial) and is provided in the *OSF Data Supplement*.

### Phylogenetic analyses of molecular data

PartitionFinder v2.1.1 (Lanfear et al., 2016) was used to select a genetic-data partitioning scheme. Input data blocks were defined by gene and by codon position. Settings specified GTR+I+G substitution models, a greedy algorithm to explore combinatorial subsetting, and scheme proposal assessments by the Bayesian Information Criterion (BIC). The optimal scheme recommended 18 partitions. The *OSF Data Supplement* includes PartitionFinder (PF) input settings and results.

Bayesian phylogenetic analyses were performed with MrBayes v3.2.7 MPI (Altekar et al., 2004; Ronquist et al., 2012b). Partitioning of the DNA supermatrix followed PF results and all partitions were assigned independent (unlinked) GTR+I+G models. We began with a standard (non-clock) analysis in which tree branch lengths estimate the number of substitutions per alignment site. MCMC settings included: 2 runs, 4 chains per run, 4 attempted swaps per generation, 50M generations, sampling in 1k generation increments, and 10M generations discarded as burn-in. We assumed a basal split leading to the Paenungulata and Afroinsectiphilia lineages (*e.g*., Esselstyn et al., 2017; Meredith et al., 2011) and applied a rooting constraint for this bifurcation. The post-burn-in tree distribution was summarized with the MrBayes option for majority rule plus all compatible groups (*i.e*., sumt contype = allcompat). The average standard deviation of split frequencies (ASDSF) was 0.000929 and the estimated sample sizes (ESS) for all substitution model parameters and per partition rate multipliers were >973. We interpret diagnostic values for ASDSF < 0.01 and minimum ESS > 200 as evidence of topological convergence and sufficient sampling of parameter space.

Next, a time-scaled (clock) analysis was run using the same supermatrix, partitioning scheme, and substitution models. Fifteen fossil taxa were added to this dataset and each was coded with all null (?) character scores. These additions represent the oldest extinct taxa that are, in our opinion, securely attributable to select stem lineages within the afrotherian clade. We updated this previously-published suite of fossil calibrators (Heritage, Seiffert & Borths, 2021) to reflect a new age estimate for *Priscosiren atlantica* (occurrence in Puerto Rico), a new age estimate for taxa from the Chambi Site in Tunisia, and—given our revised age estimate for *Prorastomus sirenoides*—substituting the “CBI-1-542 Sirenian” as the oldest known stem sea cow (see the *OSF Data Supplement* for details of these revisions). Table S1 summarizes the updated set of fossil calibrators. Extensive references concerning the geologic ages and phylogenetic placements of these fossil taxa are given in Heritage, Seiffert & Borths (2021). In the analysis settings, the ages of extant taxa were fixed to zero, recently extinct taxa were fixed to the approximate ages of the source specimens that yielded DNA sequences, and null-data fossil taxa were fixed to the ages listed in Table S1. The topology for this time-scaled analysis was constrained to the result from the standard analysis and was modified by appending fossil calibrators to their respective stem lineages. These added fossils inform hard minimum ages for ten nodal calibrations. These nodes are: crown Tethytheria (minimum age of 56 Ma *via Phosphatherium*), crown Sirenia (minimum age of 29.5 Ma *via Priscosiren*), the *Mammut-Loxodonta* split (minimum age of 25.8 Ma *via Losodokodon*), the *Elephas-Mammuthus* split (minimum age of 5.5 Ma *via Mammuthus subplanifrons*), the *Palaeoloxodon-Loxodonta* split (minimum age of 6.5 Ma *via Loxodonta* sp. indet.), the *Dendrohyrax-Procavia* split (minimum age of 6 Ma *via Dendrohyrax samueli*), the *Dendrohyrax-Heterohyrax* split (minimum age of 10.4 Ma *via Heterohyrax auricampensis*), crown Afroinsectivora (minimum age of 56.8 Ma *via Todralestes*), crown Macroscelidea (minimum age of 25 Ma *via Oligorhynchocyon*), and crown Afrosoricida (minimum age of 32.1 Ma *via Eochrysochloris*). Node calibrations applied truncated normal distributions (TND) with location parameter values corresponding to the ages listed above. Unbounded maximum ages were softly constrained with the spread parameter (SD = 5). We identify *Eritherium azzouzorum* (Table S1) as the oldest fossil species that can be confidently placed within crown Afrotheria and used the age of this taxon, plus a modest 0.5 million years, to set a hard minimum age for the tree root (TND locations = 60.5). Like all other node calibrations, the maximum root age was treated as unbounded but softly constrained (SD = 5). A phylogenetically-informed “clockrate” prior was derived using a previously-published (Gunnell et al., 2018) R language (R Core Team, 2019) script. With roughly 100 extant afrotherian species (IUCN, 2021), the DNA supermatrix samples about half of the clade’s modern biodiversity. Accordingly, we populated the “sampleprob” parameter of the Fossilized Birth-Death (FBD) model with a value of 0.5. MrBayes defaults were kept for all other FBD settings. All proposals that contain the “tau” parameter were disabled. MCMC settings included: 2 runs, 4 chains per run, 4 attempted swaps per generation, 50M generations, sampling in 1k generation increments, and 10M generations discarded as burn-in. Again, the “allcompat” option was used to summarize the post-burn-in tree distribution. ESS values for all substitution model parameters and per partition rate multipliers were >273.

### Morphology dataset

The principal morphological character-taxon matrix used in this study is an adapted version of the Vélez-Juarbe & Wood (2018) matrix. That dataset expands and updates the matrices previously used by Springer et al. (2015), Vélez-Juarbe, Domning & Pyenson (2012), and Domning (1994). We removed *Cornwallius sookensis* (order Desmostylia) from this matrix because, in our opinion, desmostylian taxa have been convincingly placed as perissodactyls and are not paenungulate afrotherians (Cooper et al., 2014; Rose et al., 2019). The modified matrix includes 60 sirenian taxa (living and fossil), the early proboscidean *Phosphatherium escuillei*, and 83 scored characters. In an effort to optimize phylogenetic signal (Wiens, 2000), we treated 18 multistate traits as ordered—the states of these characters were designed as ordered transformation series and were treated as such in the Springer et al. (2015) assessment.

The sirenian species *Hydrodamalis spissa* is potentially important for our biogeographic model but is absent from the Vélez-Juarbe & Wood dataset. *H. spissa* has been proposed as the immediate sibling-species of *Hydrodamalis gigas*, is slightly older than *H. gigas*, and is exclusively known from occurrences in Japan. To prepare to incorporate *H. spissa* into our study, we modified the character-taxon matrix of Furusawa (2004) by pruning that dataset’s taxon sample to include only *H. spissa* and the five species also present in the Vélez-Juarbe & Wood matrix. Bayesian phylogenetic analysis of morphological data includes likelihood correction for ascertainment bias which presupposes coded variability in each character column. Accordingly, we removed all invariant character columns which reduced this matrix to 28 characters (all treated as unordered). Independent pilot analyses of the modified Vélez-Juarbe & Wood and Furusawa matrices found no topological incongruence—this suggested that matrix concatenation would effectively add *H. spissa* to our study with little influence on the proposed relationships of other included taxa.

The fossil petrosal “CBI-1-542” from Chambi, Tunisia represents an additional sirenian taxon that can inform key aspects of our phylogenetic and biogeographic models. To add this specimen to our study, we followed the approach described above—starting with the character-taxon matrix from Benoit et al. (2013), reducing the taxon sample to CBI-1-542 plus six species present in the Vélez-Juarbe & Wood matrix, and then removing all invariant character columns. The resultant modified matrix is 17 characters (all treated as unordered). A pilot analysis of these data suggested that concatenation with the modified Vélez-Juarbe & Wood matrix would integrate CBI-1-542 into our study without introducing topological conflict.

With important implications for the ancestral biogeographic pattern of the earliest sirenians, we also added the “SN102” Senegalese prorastomid to the dataset. This specimen has been described as generically distinct (Hautier et al., 2012) from other prorastomids—but the limited material available at this time has inhibited taxonomic designation below the family rank and has deterred inclusion in morphological character matrices. We accommodated SN102 in our dataset by constraining it to a Prorastomidae clade (*i.e*., *Prorastomus* + *Pezosiren* + SN102), by incorporating the specimen’s geologic age and geographic provenance, and by neutral (*i.e*., null) representation for all anatomical coding. Therefore, our analyses will place SN102 among other prorastomids in a position wholly informed by temporospatial signal.

Concatenations of the three morphology matrices were performed with Mesquite v3.61 (Maddison & Maddison, 2019). Our objective was to set up analyses in which the morphology supermatrix can be partitioned in correspondence to the positions of the original matrices, with each assigned an independent Markov model. We attempted to balance the number of characters in each partition by matrix duplications (1× Vélez-Juarbe & Wood; 3× Furusawa; 5× Benoit et al.) which yielded a narrow range of 83–85 character positions per partition. With only a few characters in the original matrices that inform the phylogenetic positions of *H. spissa* and CBI-1-542, this approach effectively upweights that signal so that variable MCMC topological proposals for these two taxa are associated with larger likelihood differentials, thereby resulting in less frequent acceptance of placements that have lower likelihood values.

### Temporal and geospatial datasets

The geologic ages of included fossil sirenians were compiled from primary literature sources. We attempted to incorporate the most recent information concerning radiometric dates, magnetostratigraphy, biozonation, and other dating methods. In some cases, data from recent studies require important revisions to formerly proposed ages. For example, a 2018 assessment of the Yellow Limestone Group in Jamaica (Gold et al., 2018)—which draws from multiple lines of evidence—found that the Stettin “member” is early-to-middle Lutetian and not terminal Ypresian as previously reported (Savage, Domning & Thewissen, 1994). The implications of this revision are that *Prorastomus sirenoides* is probably not the earliest known sea cow taxon and that the oldest fossil evidence of Sirenia is more likely from North Africa than the Caribbean. Extensive discussion and supporting references for this dataset are given in the *OSF Data Supplement*.

For each fossil taxon in the morphology matrix, we also collected WGS84 decimal geocoordinates for occurrence localities. Most of these data were sourced from the aggregator GBIF (GBIF, 2022) and originated from museum specimen records or the primary literature. In a few cases, longitude and latitude were estimated *de novo* using literature accounts of the localities in conjunction with Google Earth Pro v7.3.4 (Google Earth, 2021). The *OSF Data Supplement* reports the details of the geocoordinates dataset (also summarized in Table S2). Where a taxon is known from only a single occurrence (*e.g*., *Pezosiren portelli*), we simply recorded the geocoordinates of the type locality. If a taxon is known from multiple occurrences, but all localities are relatively nearby and share a single age range (*e.g*., *Caribosiren turneri*), we chose a single representative locality and recorded the geocoordinates of that site. If a taxon is known from multiple localities that have substantial geographic separation (*e.g*., *Priscosiren atlantica*, which is known from both Puerto Rico and South Carolina), we recorded separate geocoordinates for each locality. For multiple locality taxa for which sites are estimated with different age ranges (*e.g*., *Crenatosiren olseni*), we coupled the geocoordinates at each locality with their own upper and lower age bounds.

### Biogeographic coding

**Figure 2:**
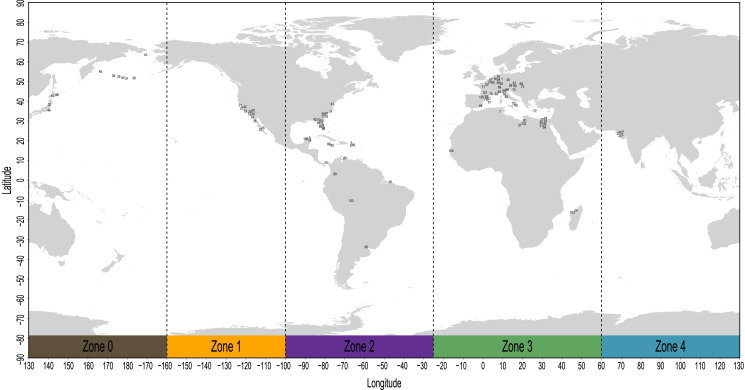
Mapped localities of fossil occurrences included in this study. Plotted numbers correspond to map IDs in Table S2. Vertical dashed lines identify bounds of the longitudinal zones used for coding biogeographic character. Adapted base map from Mapswire.com *(CC-BY 4.0)* (https://mapswire.com/).

The geospatial distribution of all fossil sirenians in the dataset was plotted using the compiled geocoordinates discussed above (Fig. 2). From this plot, we identified five contiguous longitudinal zones that reasonably correspond to the patterns of mapped localities. Zone codes and their definitions are: (0) 130 to −160 degrees, Far East Asia and Beringia *sensu lato*; (1) −160 to −100 degrees, western North America; (2) −100 to −25 degrees, eastern North America, South America, and the Caribbean; (3) −25 to 60 degrees, Europe and Africa; and (4) 60 to 130 degrees, Pan Asia (excluding the Far East). All localities in the geocoordinates dataset were binned into this zonation scheme and all fossil taxa were coded accordingly. Pilot analyses of the morphology dataset prior to biogeographic coding found no evidence of fossil taxa from South Asia (Zone 4) that inform lineages that later moved eastward into Far East Asia or Beringia (Zone 0). Therefore, we elected to define this multistate character as ordered to capture spatial contiguity. The geographic distributions of all three extant species of manatees (genus *Trichechus*) fit discretely into these bins and were coded with their corresponding zones. The extant species *Dugong dugon*—with a probable lineage origin in the Caribbean or northern-adjacent west Atlantic (Domning, 2001b; Vélez-Juarbe, 2012), a possible ancestral migration westward into the Pacific (Vélez-Juarbe, 2012), and a very large modern geographic range that spans our Zones 0&4&3—was coded with a null datum to avoid potentially confounding the contiguity of the ordered transformation series. We used Mesquite v3.61 to append this biogeographic character to the morphology supermatrix.

### Phylogenetic analysis of morphological data

Time-scaled phylogenetic analysis of the morphology [+ biogeographic character] supermatrix applied a BTD strategy (Ronquist et al., 2012a). The matrix was subsetted into four partitions: (1) Vélez-Juarbe & Wood; (2) Furusawa; (3) Benoit et al.; and (4) Geographic Zone. Each partition was assigned an independent Markov model and variable coding was enabled to accommodate ascertainment bias. Settings for character ordering followed the descriptions above.

We split multiple locality taxa into *n* operational taxonomic units (OTUs) if occurrence localities had *n* different age ranges. For example, the included occurrences for *Metaxytherium floridanum* from Florida are from a more southern site in Hillsborough County (age range 11.95–14.6 Ma) and a more northern site in Alachua County (age range 9.3–11.95 Ma). In this case, the *M. floridanum* row in the matrix was duplicated to yield two OTUs. Analysis settings constrained both of these OTUs to an exclusive clade. The OTU associated with the oldest age range retained the original character coding while the OTU with the younger age range was recoded with all neutral character scores. This approach assumes that the anatomy captured by the matrix had evolved by the earlier occurrence. Furthermore, the morphological distances among all taxa in the matrix—which are metrics incorporated into BTD analyses—are forced to be calculated between earliest occurrences, while later occurrences (represented by neutral data) do not bias the rates derived from these distances. In cases where a taxon was split into more than two OTUs, the algorithm will arbitrate relationships among the constrained clade based only on the ages of the occurrence localities.

For tip age priors, uniform distributions were assigned with upper and lower bounds corresponding to the geologic age ranges of each OTU. The ages of extant taxa were fixed to zero. A “molecular scaffold” was applied as a partial constraint where the relationships of extant sirenians (plus Steller’s sea cow) were assumed to correspond to the topology from our analyses of the DNA supermatrix. With all four extant sirenian species represented in this dataset, the “sampleprob” parameter of the FBD model was set to 1. All other FBD settings and priors retained MrBayes defaults. The “clockrate” prior was set to an uninformative (*i.e*., approximately flat) distribution. The fossil proboscidean *Phosphatherium* was constrained as the ultimate outgroup taxon. This outgroup is also the oldest OTU in the dataset and therefore provides a hard minimum age (56 Ma) for the tree root. We used this age directly to populate the location parameters of the unbounded TND that was assigned as the “treeage” prior. Deeper ages sampled from this distribution were softly constrained by the spread parameter (SD = 5).

MCMC settings included: 4 runs, 8 chains per run, 8 attempted swaps per generation, 100M generations, sampling in 1k generation increments, and 10M generations discarded as burn-in. The post-burn-in tree distribution was summarized with the “allcompat” option. The ASDSF for this analysis was 0.002849 and the ESS for all substitution model parameters were >4,145. The posterior distribution of tree-tip ages was extracted and summarized with the “*MrBayes - Parse Tip Ages*” toolkit for R (Heritage, 2022a).

### Phylogenetic analysis of the total evidence dataset

The DNA supermatrix and morphology [+ biogeographic character] supermatrix were concatenated using Mesquite v3.61. BTD analysis of this total evidence (TE) dataset allows us to incorporate rates of molecular evolution within Sirenia—in conjunction with fossil “tip calibrations” within the order and multiple external “node calibrations” among non-sirenian clades.

We began by deriving a phylogenetically-informed distribution for the “clockrate” prior. This step requires a non-clock tree estimated from the TE supermatrix. To this end, we grafted the Afrotheria clock tree from the DNA analysis with the Sirenia clock tree from the morphology [+ biogeographic character] analysis and then stripped the time-scaled branch lengths. We assumed that the resultant topology was a reasonable starting estimate. Non-clock Bayesian analysis of the TE dataset proceeded using this topology as a complete set of hard constraints and applied the same models, partitioning scheme, and character ordering used in the previous assessments of the component matrices. All proposals that contain the “tau” parameter were disabled. This strategy effectively fits “substitutions per site” branch lengths to the fixed topology using the TE supermatrix. MCMC settings included: 2 runs, 4 chains, 4 attempted swap per generation, 35M generations, sampling in 1k generation increments, and 10M generations discarded as burn-in. Next, we extracted the median tip ages from the Sirenia clock tree and combined them with the tip ages of non-sirenians from the Afrotheria clock tree. Lastly, the median root age from the Afrotheria clock tree was recorded as an initial estimate for the TE tree root. Using the non-clock TE tree, a full set of tip age estimates, and a root age estimate, we ran the previously reported R script (Gunnell et al., 2018) which fits several distributions to these data and assesses candidate models with BIC scores. In this case, the optimal model (*i.e*., with the lowest BIC value) was lognormal (mean = −4.834237, sd = 0.377004). This distribution, and its parameter values, were used directly as the “clockrate” prior for the TE analysis.

For the final phylogenetic assessment, the TE supermatrix was subsetted into 18 partitions with unlinked GTR+I+G models (corresponding to the previous analyses of molecular data) and four additional partitions with unlinked Markov models (corresponding to the previous analysis of morphological + biogeographic data). For partitions 19–22, we enabled variable coding and set character ordering as previously described. Drawing from the clock analysis of the DNA supermatrix, we replicated the fixed tip ages for non-sirenian taxa and applied the same nine nodal calibrations that fall outside of Pan-Sirenia. Tip age priors for fossil sirenian OTUs were assigned uniform distributions identical to those discussed above. Tip ages for extant sirenians were fixed to zero. The “sampleprob” of the FBD model was set to 0.5 but all other FBD settings retained MrBayes defaults. The topological relationships among non-sirenian taxa were constrained to reproduce the results of the preceding analyses. The distribution assignment for the “treeage” prior (*i.e*., the crown Afrotheria split) replicated the settings used in the clock analysis of the DNA supermatrix. MCMC settings included: 4 runs, 8 chains per run, 8 attempted swaps per generation, 100M generations, sampling in 1k generation increments, and 10M generations discarded as burn-in. The “allcompat” option was used to summarize the post-burn-in tree distribution. For this analysis, the ESS for all substitution model parameters and per partition rate multipliers were >210 and the ASDSF was 0.00616. The posterior distribution of tree-tip ages was parsed and summarized as previously described.

### Ancestral state reconstruction of discrete traits

To characterize the patterns of trait evolution against the context of our time-scaled TE phylogenetic hypothesis, we performed ancestral state reconstruction (ASR) of discrete traits using the Bayesian toolkit MBASR (Heritage, 2021b). As a preparatory step, the TE time-tree was pruned by removing all non-sirenian taxa except *Phosphatherium*. We set-up assessments for all 83 characters in the modified Vélez-Juarbe & Wood morphology matrix plus our geographic zone character. Traits in the other two morphology matrices were excluded because limited taxon sampling in those datasets poorly inform reconstructions for most nodes in the larger TE tree. Analysis settings specified 3,500 samples per character. Results include node probability tables for each trait in which the marginal likelihoods of all character states are reported for all nodes in the pruned TE tree. In addition, a tree plot is produced for each trait in which coded states are indicated at tree tips and marginal likelihoods for all reconstructed states are represented as pie-charts that overlay tree nodes. ASR analysis input and output are provided in the *OSF Data Supplement*.

### Ancestral state reconstruction of continuous geocoordinates

Ancestral biogeography was modeled as a continuous variable using BayesTraits v4.0.0 (Meade & Pagel, 2022) and the “*BTAGR*” toolkit for R (Heritage, 2021a). To prepare for this analysis, all non-sirenian taxa except *Phosphatherium* were pruned from the TE time-tree. Next, we retrieved the currently recognized species range polygons (IUCN, 2021) for each of the four extant sea cow species and used farthest point sampling to “evenly” sample *n* points from their distributions (*Trichechus n* = 30 per species; *Dugong n* = 70). These points were used as representative geocoordinates for their respective species.

Our assessment followed a two-pass strategy. For the first pass, we used the Sirenia + *Phosphatherium* version of the TE time-tree where some tips bundle two or more occurrence localities. Geographic centroids were calculated for each multi-locality tree tip. Next, each tip in the tree was matched with either its unique geocoordinate pair (Table S2) or its calculated geocentroid. BTAGR analysis was run with variable rates enabled, tectonic plates at time slice = 0, and MCMC *n* samples = 2,500. The software automatically removes burn-in and poor likelihood generations before writing the analysis trace and summary. The post-analysis ESS range of sampled parameters was 1,371–2,008. We interpret a minimum ESS >200 to indicate sufficient sampling and run length. Input files, settings, and full results are provided in the *OSF Data Supplement*.

For the second-pass, the input tree was modified by splitting multi-locality tips into their individual occurrences. To do so, we applied the following strategies: (a) Where a single tip bundles *two* localities, the terminal was bifurcated and each of the two new tips retained their original age. The age of the newly created tree node was estimated by calculating the geographic distance between the two localities and then scaling that distance to units of time. The scaling factor was determined by first extracting the age difference between the original single tip and its parent node (from the TE time-tree), and then by calculating the geographic distance between the single tip’s geocentroid and the parent node’s mean geocoordinates (reconstructed in the first-pass analysis). (b) Where a single tip bundles *three or more* localities, the geocoordinates for all of the occurrences were used to calculate a geographic distance matrix for the set. Neighbor-joining phylogenetic clustering was applied to this distance matrix to produce an unrooted tree. Next, we retrieved the parent node’s mean geocoordinates (reconstructed in the first-pass analysis) and used that position to identify the single locality (bundled in the corresponding TE time-tree tip) with the shortest geodistance to the parent node. The neighbor-joining tree was then rooted on the terminal branch of this closest-to-parent locality and nonparametric rate smoothing was applied to produce a flush-tipped transformation. Next, the branch lengths in this geodistance tree were scaled to units of time using the scaling factor strategy discussed above. Finally, the rooted neighbor-joining time-tree was grafted to the corresponding TE time-tree tip in a manner that shortened the original terminal branch by the amount necessary to produce new tip ages equivalent to the original tip age.

After modification, the input tree used for the second-pass analysis included 106 tips for fossil sirenians and each corresponds to a single georeferenceable locality (Table S2). Additionally, individual geocoordinate pairs sampled from extant species ranges were each matched with unique tree tips. BTAGR analysis settings replicated those used in the first-pass assessment. The post-analysis ESS range of sampled parameters was 608–992. Using the summarized results, the software plots an equirectangular map projection onto which it places (a) points corresponding to the input geocoordinates of tree tips, and (b) points corresponding to the mean longitude and latitude of reconstructed tree nodes. Tree branches are then drawn as geodesic arcs to connect the network of plotted points. With the calculated means and covariances of geocoordinates for each tree node, confidence ellipses were drawn to express uncertainty in nodal reconstructions. We selected 68% ellipses to denote a geographic area within which the majority (roughly two-thirds) of spatial estimates were concentrated. Input files, settings, and full results are provided in the *OSF Data Supplement*.

### Lineage counts through time

To quantify the diversity of sirenian lineages as a function of time, we used the “*Time-Slice LTT*” toolkit for R (Heritage, 2022b). To prepare for this assessment, the TE time-tree was pruned by removing all non-sirenian tips. Next, we identified sirenian species represented by multiple terminal branches (*i.e*., those with more than one occurrence age) and retained only the terminal branches that correspond to the latest occurrences. Starting from this modified tree, branches were subsetted according to their geographic reconstructions. Where discrete trait ASR of parent and child nodes (or tips) were both the same geographic zone, we considered their connecting branch to be confidently assignable to a single zone. Where parent and child nodes were reconstructed in different zones, we assumed that the transition occurred along their connecting branch and excluded it from unambiguous geographic subsets. Analysis settings specified lineage counts at half-million-year increments and quantifications were run independently for the full tree and select subsetted zones. All input files and settings are provided in the *OSF Data Supplement*.

### Definition of terms

In the context of this manuscript, we use the terms “Tethys” and “Tethys Sea” to refer to the Paleogene + Aquitanian (66-20 Ma) body of water—situated between northern Afro-Arabia and southern Europe—that connected the Atlantic Ocean to the west of the Iberian Peninsula with the proto-Indian Ocean to the east of the Arabian Plate. This is equivalent to the proto-Mediterranean + Paratethys of Torfstein & Steinberg (2020) (see their Fig. 1).

## Results

### Analyses of the DNA supermatrix

**Figure 3:**
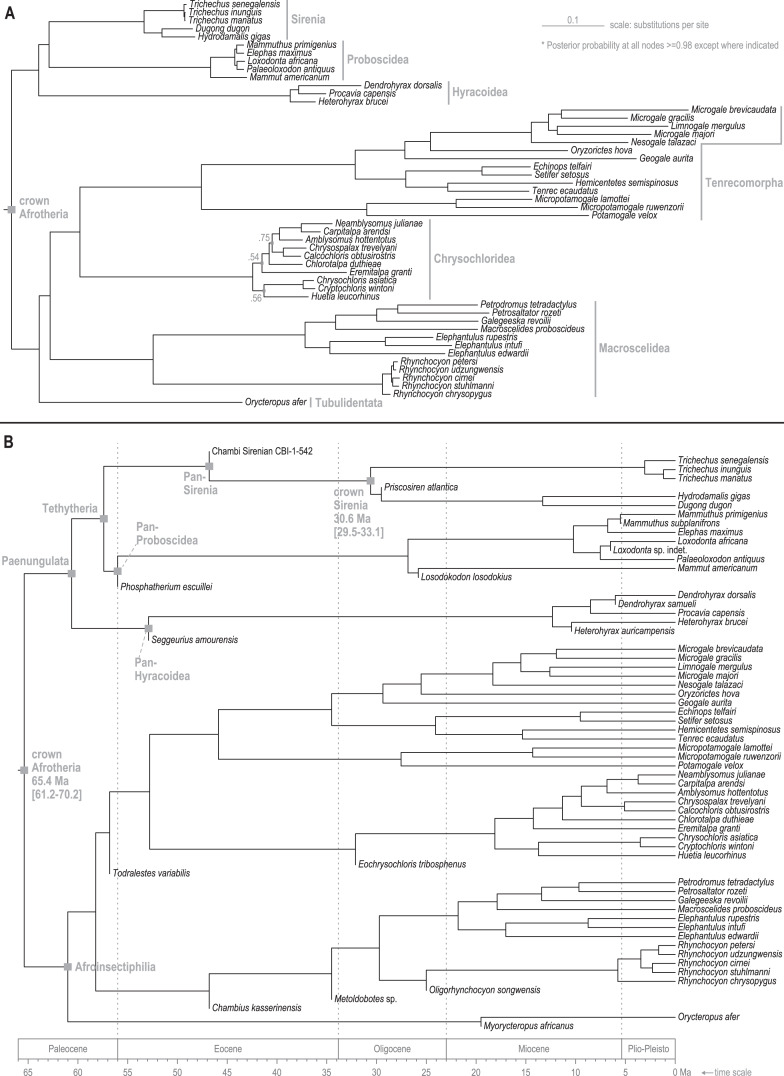
Consensus trees from Bayesian phylogenetic analyses of the DNA supermatrix. (A) Standard. (B) Time-scaled.

Standard (non-clock) Bayesian phylogenetic analysis of the molecular dataset yielded posterior probability (PP) values for lineage splits that were very high (>=0.98) throughout tree, with the exception of three nodes nested within the Chrysochloridea group (Fig. 3 and *OSF Data Supplement*). The basal split at the crown Afrotheria node into the Paenungulata and Afroinsectiphilia lineages was a rooting assumption of the analysis that was based on the results of several previous studies (*e.g*., Esselstyn et al., 2017; Meredith et al., 2011). The recovered relationships among the major Afroinsectiphilia clades—namely (Tubulidentata, (Macroscelidea, (Chrysochloridea, Tenrecomorpha)))—were identical to the results of many molecular studies that contain compatible taxa (*e.g*., Heritage et al., 2020; Kuntner, May-Collado & Agnarsson, 2011; Springer et al., 2015). Within Paenungulata, the recovery of a Tethytheria group (*i.e*., Sirenia + Proboscidea) to the exclusion of Hyracoidea is congruent with morphological studies (*e.g*., Novacek, 1992), some assessments of large-scale DNA sequence data (*e.g*., Springer et al., 2015; Poulakakis & Stamatakis, 2010), and a recent analysis of genomic-scale rare indel events (Schull et al., 2022). Within Sirenia, the recovery of *Hydrodamalis gigas* as sister to *Dugong dugon*, and the placement of *Trichechus senegalensis* as basal among the three *Trichechus* species, was expected given the previously published molecular studies that sample these taxa (de Souza et al., 2021; Springer et al., 2015). Among the major afrotherian clades, “substitutions per site” branch lengths were longest within Tenrecomorpha, indicating higher rates of molecular evolution relative to the other groups.

The time-scaled (clock) phylogenetic analysis of the same DNA supermatrix (Fig. 3 and *OSF Data Supplement*) yielded divergence age estimates that closely reflect those reported by Heritage, Seiffert & Borths (2021). Compared to that study, ours varied in sampled taxa but employed a highly similar fossil calibration strategy. For select nodes, median age estimates with 95% highest density intervals (HDIs) were: crown Paenungulata (60.6 Ma, 57.3-65.1 Ma), crown Tethytheria (57.4 Ma, 56.0-60.3 Ma), crown Sirenia (30.6 Ma, 29.5-33.1 Ma), crown Dugongidae (13.3 Ma, 4.7-19.0 Ma), and crown Trichechidae (3.1 Ma, 1.0-6.0 Ma). In geologic terms, the Trichechidae-Dugongidae split was estimated to have occurred during the early Oligocene (Rupelian), the *Dugong*-*Hydrodamalis* split during the late middle Miocene (Serravallian), and the earliest split among the extant *Trichechus* species during the late Pliocene (Piacenzian). The *OSF Data Supplement* includes median age estimates and HDIs for all nodes in the molecular time-tree.

### Analyses of the total evidence v. morphology [+ biogeography] supermatrices

**Figure 4:**
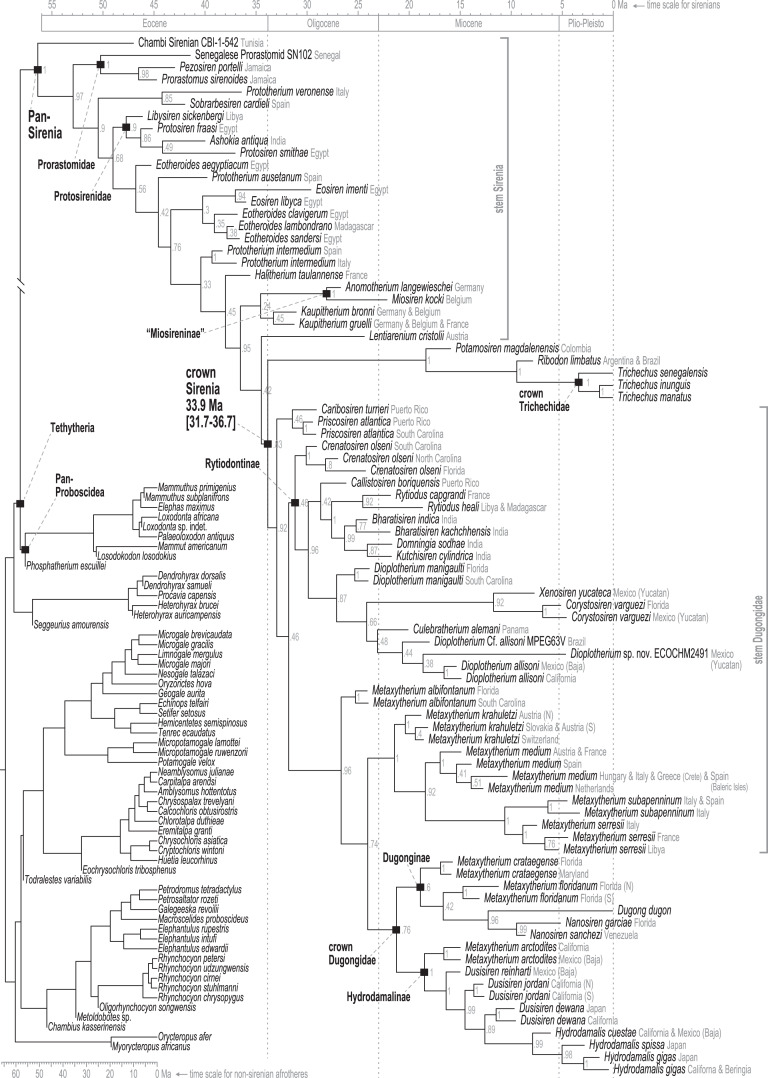
Consensus tree (allcompat) from the time-scaled Bayesian tip-dating analysis of the total evidence supermatrix.

Time-scaled Bayesian phylogenetic assessments of the total evidence (TE) and morphology [+ biogeographic character] (M+B) datasets yielded remarkably similar trees (Fig. 4 and Fig. S1). Both estimates recovered an exclusive clade containing *Dugong* and *Nanosiren* with strong support (PP >= 0.96). In the M+B result, the *Dugong*-*Nanosiren* stem connects as sister to a clade containing all *Metaxytherium, Dusisiren*, and *Hydrodamalis* species. However, the TE analysis (which differed by the inclusion of molecular data) placed the *Dugong*-*Nanosiren* group in a more nested position and within a clade of Miocene *Metaxytherium* species from the west Atlantic—thereby excluding European and North African *Metaxytherium* species, and the Oligocene west Atlantic *M. alibifontanum*, from crown Dugongidae (*sensu* this study). Otherwise, the topologies resulting from the two analyses were perfectly congruent. Despite this difference, the estimated age ranges (*i.e*., HDIs) of tips throughout the TE and M+B time-trees were highly correlative (Table S2).

Because the key difference between the two hypotheses concerns the placement of the extant species *Dugong dugon*, there is also notable variation in the age estimates for the crown Dugongidae node. The less nested position of *D. dugon* (M+B analysis; Fig. S1) placed the crown dugongid split in the mid-Oligocene (27.4 Ma, 24.7-30.5 Ma) while the more nested position (TE analysis; Fig. 4) shifted this divergence estimate to several million years later, during the earliest Miocene (21.2 Ma, 18.3-24.5 Ma).

Stepping-stone sampled optimizations of the TE supermatrix against the TE and M+B time-trees (*OSF Data Supplement*), yielded marginal log-likelihood values of −215136.32 and −215335.74, respectively. Thus, the TE tree was evaluated as the more likely hypothesis with Ln(BayesFactor) = 199.42 (Basu & Chib, 2003). We interpret this value as decisive evidence (*i.e*., odds >> 100:1) in favor of the better-fitting model (Jarosz & Wiley, 2014). Given the relative statistical powers of both hypotheses (BayesFactor-evaluated), and considering that most attributes of these time-trees are quite similar, in what follows we focus our reporting on the results from the TE analysis. Median age estimates and accompanying HDIs for all nodes in both trees are included in the *OSF Data Supplement*.

### Pan-Sirenia origins and early stem sea cows

**Figure 5:**
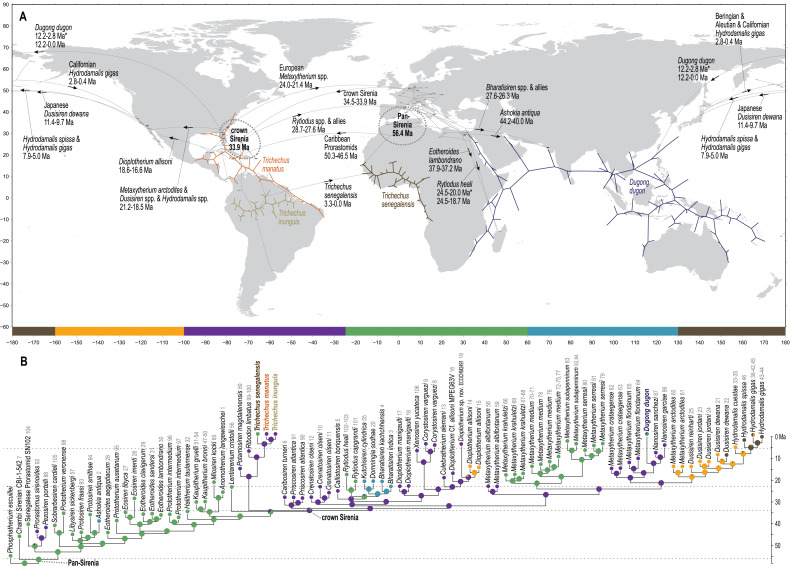
Results from ancestral state reconstructions of biogeography against the total evidence phylogenetic tree. (A) Continuous trait geocoordinates-based reconstruction—dotted loops around Pan- and crown Sirenia indicate 68% confidence ellipses of nodal reconstructions. (B) Discrete trait zone-based reconstruction—pie charts summarize marginal likelihoods of trait states at each node and numbers that follow tip names correspond to map IDs from Table S2. Adapted base map from Mapswire.com *(CC-BY 4.0)* (https://mapswire.com/).

The Chambi petrosal CBI-1-542 (Benoit et al., 2013) was recovered as the most basal stem member of Pan-Sirenia (Fig. 4) and the lineage leading to this taxon was estimated to have diverged from all other sea cows ~56.4 Ma (terminal Paleocene). CBI-1-542 is also the oldest known fossil sirenian, with a median estimated tip age of ~47 Ma—near the beginning of the middle Eocene (*i.e*., during the early Lutetian substage). The geospatial provenance of this specimen helps to situate the reconstructed Pan-Sirenia node in northwest Africa (Fig. 5). The trans-Atlantic dispersal of prorastomids to the Caribbean was estimated to have occurred between ~50.3 and ~46.5 Ma, during the early Eocene (Ypresian) or early middle Eocene (Lutetian), with the common ancestor of the Jamaican prorastomids situated in the Greater Antilles.

In contrast to the large polytomy of stem sirenians recovered by the parsimony analysis of Vélez-Juarbe & Wood (2018), our analysis identified some well-supported splits within the branching pattern of the stem group (Fig. 4). The clade that excludes Prorastomidae (*i.e*., *Prorastomus* + *Pezosiren* + SN102) and the Chambi petrosal was recovered with strong credibility (PP = 0.9). Most of the subsequent evolution along the stem lineage, from ~53 Ma to ~40.6 Ma (Ypresian into the early Bartonian), was reconstructed as having been centered in the Tethys Sea, between southern Europe and northern Africa (Fig. 5).

Further along the stem, our results include a well-supported (PP = 0.85), early-diverging clade of southern European species which includes *Prototherium veronense* (Italy; de Zigno, 1875; Sickenberg, 1934) and *Sobrarbesiren cardieli* (Spain; Díaz-Berenguer et al., 2018). This group has two unambiguous synapomorphies (in-text format is “character.state, definition”; see full ASR results in the *OSF Data Supplement*; 29.1, cranial portion of squamosal extends to temporal crest; 60.1, cross-section of I^1^ crown lens-shaped, with sharp anterior and posterior edges). This result suggests that *Prototherium* is polyphyletic, as the two other included congeners—*Prototherium ausetanum* (Spain; Balaguer & Alba, 2016) and *Prototherium intermedium* (Spain and Italy; Bizzotto, 1983)—were placed in more advanced positions of the tree. Although the range of PP values (0.33–0.76) for this focal portion of the early stem was low to moderate, *Prototherium* monophyly is incompatible with the optimal credibility consensus tree.

The next crownward divergence from the stem lineage leads to a well-supported (PP = 0.9) Protosirenidae clade, which was estimated to have arisen ~47.8 Ma, and here includes *Libysiren sickenbergi* as its most basal member. Unlike previously published parsimony trees (*e.g*., Springer et al., 2015; Vélez-Juarbe & Wood, 2018), we note that BTD analysis recovered this clearly-defined taxonomic family as monophyletic, and with high credibility. *Ashokia antiqua* from India (Bajpai et al., 2009) is nested within *Protosiren*, being more closely related to *Protosiren smithae* (Domning & Gingerich, 1994) than the older form *Protosiren fraasi* (Abel, 1904), both of which are from Egypt. The *Ashokia* + *Protosiren* association is also robust (PP = 0.86). These results do not take into account sea cow specimens from the Eocene of North America that have been interpreted as either protosirenids (Domning, 1982; Beatty & Geisler, 2010) or *Eotheroides* (Zalmout & Gingerich, 2012) as these fossils have not yet been scored for phylogenetic analysis (see Discussion). The dispersal of the *Ashokia* lineage from the eastern Tethys to South Asia was estimated to have occurred between ~44.1 and ~40 Ma., well before the closing of the Tethys seaway (Torfstein & Steinberg, 2020). Protosirenidae is supported by five unambiguous synapomorphies (27.1, post-tympanic process of squamosal less salient, ends nearly in line with the ventral squamosal border; 31.1, outline of zygomatic process of squamosal regularly or irregularly rounded anteriorly, with non-triangular, sometimes inflated cross-section; 39.1, lacrimal without foramen, but still large; 44.0, pterygoid fossa absent; 49.2, mandibular symphysis wider, with rows of alveoli separated by a concavity broader than one alveolus).

The *Eotheroides* type species, *Eotheroides aegyptiacum* (Owen, 1875), is the next crownward branch off the main stem, and is divorced from three other recently named congeners—*Eotheroides clavigerum* and *Eotheroides sandersi* from Egypt (Zalmout & Gingerich, 2012) and *Eotheroides lambondrano* from Madagascar (Samonds et al., 2009)—all of which are geologically younger than the type. This latter triad was recovered as monophyletic and was weakly placed as the sister clade of *Eosiren* species. Nonetheless, a moderately supported (PP = 0.76) preceding split contests the monophyly of the *Eotheroides* genus. Another isolated monospecific branch off the main stem leads to *Prototherium ausetanum* from Spain (Balaguer & Alba, 2016) and intervenes between *E. aegyptiacum* and the five-species clade of *Eosiren* and *Eotheroides* sea cows. This latter group is supported by one unambiguous synapomorphy (39.1, lacrimal without foramen, but still large). The placement of *Eosiren libyca* (Andrews, 1902) as sister to *Eosiren imenti* (Domning et al., 1994) is highly credible (PP = 0.94), but we detected no unambiguous synapomorphies along their joint stem. Our analyses reconstructed the dispersal of the *E. lambondrano* lineage from the eastern Tethys into the Indian Ocean (and ultimately to Madagascar) as having occurred sometime between ~37.9 and ~37.2 Ma.

Of all species in the TE supermatrix, *E. lambondrano* has the least temporal control (*OSF Data Supplement*), with an age prior spanning from the base of the Lutetian (48.07 Ma) to the Eocene-Oligocene boundary (EOB, 33.9 Ma). As BTD analyses can provide age estimates for fossil localities whose ages are poorly constrained (*e.g*., Sallam & Seiffert, 2016; Campbell et al., 2021), it is of note that the TE analysis provided a median estimate of ~37.2 Ma (early Priabonian) for *E. lambondrano* (see Table S2 and the *OSF Data Supplement* for HDI ranges).

### Advanced stem sea cows

The advanced portion of the sirenian stem lineage includes seven species from Europe, the most basal of which are *Prototherium intermedium* and *Halitherium taulannense*. Both of these taxa have been previously recovered (by parsimony methods) as stem sea cows (Vélez-Juarbe, 2012; Vélez-Juarbe & Wood, 2018). However, the splits that most closely precede the crown node lead to *Kaupitherium greulli* (Voss & Hampe, 2017), *Kaupitherium bronni* (Voss & Hampe, 2017), and *Lentiarenium cristolii* (Voss, Berning & Reiter, 2016)—three species that have been proposed (again by parsimony methods) as deriving from the earliest divergences within crown Sirenia (Vélez-Juarbe & Wood, 2018). In other words, BTD analysis of the total evidence dataset placed these Oligocene species just outside of the crown clade, but parsimony analysis of a single morphological partition has positioned them just inside the group.

Miosireninae (*i.e*., *Miosiren kocki* + *Anomotherium langewieschei*) is a maximally supported clade (PP = 1) that was weakly resolved among the other advanced stem sirenians. Nevertheless, a highly credible split (PP = 0.95) from the main stem suggests that a more advanced placement of miosirenines is unlikely. This result contrasts with previously published parsimony assessments that have indicated that miosirenines are both crown sirenians and stem trichechids (Springer et al., 2015; Vélez-Juarbe, 2012; Vélez-Juarbe & Wood, 2018). Further, a rather credible crown Sirenia node (PP = 0.83) supports the exclusion of all seven of these advanced stem species from the crown group. The tree node that immediately precedes the crown split was reconstructed in northern Europe (Fig. 5), and the westward trans-Atlantic dispersal that led to crown Sirenia origins, was estimated to have occurred between ~34.5 and ~33.9 Ma (terminal Eocene).

The clade that includes crown Sirenia + the seven advanced stem species is supported by four unambiguous synapomorphies (34.1, ventral extremity of jugal lies approximately under posterior edge of orbit, but forward of jugal’s postorbital process; 62.1, second and third upper incisors, first through third lower incisors all absent; 63.2, canines absent; 64.1, some anterior premolars 1-4 absent)—while the crown Sirenia + *Lentiarenium* clade is supported by three (15.1, nasals separated in midline by frontals and/or an incisure or separated and fused with frontals; 49.3, mandibular symphysis broad, more or less rectangular, without functional alveoli; 50.3, ventral border of horizontal mandibular ramus strongly concave).

### Crown Sirenia

The divergence of Trichechidae from Dugongidae was estimated to have occurred at the Eocene-Oligocene boundary (33.9 Ma, 36.7-31.7 Ma), with the location of the crown group’s ancestral population reconstructed near the modern-day position of the Bahamas (Fig. 5). Crown Sirenia is supported by two unambiguous (but non-independent) synapomorphies (64.2, premolars 1-4 all absent; 70.2 [number of roots on] permanent premolars all absent). As mentioned above, posterior probability at the crown Sirenia split is 0.83.

### Trichechidae

Approximately the first half of the ~30.5 Ma long trichechid stem lineage is not yet represented by fossils. Although fragmentary fossils of early Miocene stem trichechids have been described (Suarez et al., 2021), the only stem trichechids in the dataset are *Potamosiren magdalenensis* from the middle Miocene of Colombia (Reinhart, 1951; Suarez et al., 2021) and *Ribodon limbatus* from the late Miocene of Argentina and Brazil (Ameghino, 1883). The successive placements of these taxa along the trichechid stem are robust (PPs = 1). The Pan-Trichechidae split was estimated to be ~18.3 Ma (early Miocene) and was reconstructed in central Colombia. Three unambiguous synapomorphies support this clade (7.1, zygomatic-orbital bridge of maxilla elevated above palate, with its ventral surface lying >1 cm above alveolar margin; 50.2, ventral border of horizontal mandibular ramus moderately and evenly concave; 55.0, horizontal mandibular ramus slender, minimum dorsoventral height <0.25× length of mandible).

The common ancestor of the three extant *Trichechus* species (*i.e*., crown Trichechidae) was estimated to be ~3.3 Ma (Pliocene) and was reconstructed in the Amazon Basin of northwest Brazil (*i.e*., within the modern geographic range of *T. inunguis*). *T. senegalensis* was recovered as the basal-most species among crown manatees—which constrains the Africa-bound trans-Atlantic dispersal of this taxon’s stem lineage to sometime after the ~3.3 Ma crown split. The *T. inunguis*-*T. manatus* split was estimated to be ~1.3 Ma (Pleistocene), and was also reconstructed in northwestern Brazil, only a few hundred kilometers north of the reconstructed crown trichechid node.

### Dugongidae and early stem dugongids

Unlike several previous studies that have explicitly used Dugongidae as a paraphyletic taxon within which Trichechidae is nested (*e.g*., Domning, 1994), here we restrict Dugongidae to those living and extinct species that are (based on the results of our analysis) more closely related to extant *Dugong* than to extant *Trichechus*. Within Pan-Dugongidae we recognize three subfamilies—Dugonginae and Hydrodamalinae are reciprocally monophyletic groups of crown dugongids, and Rytiodontinae is a clade of stem dugongids (Fig. 4).

The earliest diversification from the Pan-Dugongidae lineage leads to a clade that includes *Caribosiren turneri* (Reinhart, 1959) from the early Oligocene of Puerto Rico and *Priscosiren atlantica* (Vélez-Juarbe & Domning, 2014b) from the early Oligocene of both Puerto Rico and South Carolina. *Caribosiren, Priscosiren*, and the earliest occurrence of *Crenatosiren* (which was placed in a slightly more nested phylogenetic position; Fig. 4) were all approximately contemporaneous and potentially sympatric (Vélez-Juarbe & Domning, 2014b). *Caribosiren* and *Priscosiren* were placed as stem dugongids with high credibility (PP = 0.92). They are also the oldest known pan-dugongid taxa and therefore place a minimum constraint on the age of crown Sirenia. The Puerto Rico occurrence of *Priscosiren atlantica* is marginally older the others (median tip age = 29.45 Ma).

### Rytiodontinae

Domning (1994) included the genera *Corystosiren*, *Crenatosiren*, *Dioplotherium*, *Rytiodus*, and *Xenosiren* in the subfamily Rytiodontinae. Our analysis recovered a clade that groups these taxa but also includes the more recently described *Bharatisiren* (Bajpai & Domning, 1997), *Callistosiren* (Vélez-Juarbe & Domning, 2015), *Culebratherium* (Vélez-Juarbe & Wood, 2018), *Domningia* (Thewissen & Bajpai, 2009) and *Kutchisiren* (Bajpai et al., 2010). The divergence between Rytiodontinae and crown Dugongidae was estimated to be ~31.8 Ma (early Oligocene) and was reconstructed near northeastern Florida.

*Crenatosiren olseni* was weakly placed as the basal-most member of Rytiodontinae, with its inclusion supported by two unambiguous synapomorphies (17.1, nasal incisure at posterior end of mesorostral fossa deep and narrow, extends posterior to supraorbital process; 38.1, ventral rim of orbit does distinctly overhang the lateral surface of the jugal). The next nested node, which excludes *Crenatosiren* but groups all other rytiodontine taxa, is highly credible (PP = 0.96). With an estimated age of ~29.9 Ma, this more exclusive clade is supported by three unambiguous synapomorphies (18.2, frontal roof deeply concave or depressed, and sloping steadily ventrad to anterior margin; 36.1, preorbital process of jugal thick and robust; 60.2, cross section of I^1^ crown lozenge-shaped). Further, this node marks a major split within the subfamily that leads to two large sister clades—a group of Western Hemisphere sea cows that includes the genera *Corystosiren*, *Culebratherium*, *Dioplotherium*, and *Xenosiren*—and a group that includes *Callistosiren* (from Puerto Rico) as basally connected to a clade of Eastern Hemisphere genera, namely *Bharatisiren*, *Domningia*, *Kutchisiren*, and *Rytiodus*.

From the west Atlantic, the trans-Atlantic dispersal that led to the origin of the Eastern Hemisphere rytiodontine group was estimated to have occurred between ~28.7 and ~27.6 Ma (mid-Oligocene, near the Rupelian-Chattian boundary). The common ancestor of this clade was reconstructed just north of Egypt with descendant occurrences of *Rytiodus* species known from southern Europe (~21.8 Ma, Aquitanian, earliest Miocene) and northern Africa + Madagascar (~18.7 Ma, Burdigalian, late early Miocene). One unambiguous synapomorphy supports the Eastern Hemisphere rytiodontine clade (41.2, posterior border of palatine very deeply incised to as far forward as level of M^1^). Originating from the eastern Tethys, a subsequent eastward dispersal that gave rise to the rytiodontines of South Asia, was estimated to have occurred between ~27.6 and ~26.3 Ma (Rupelian, late Oligocene) which predates the effective closure of the Indian Ocean-Mediterranean Seaway by several million years (~21-20 Ma, Torfstein & Steinberg, 2020). Both the Eastern Hemisphere and more exclusive South Asia rytiodontine splits are highly credible (PPs >= 0.99).

The other major rytiodontine clade—*i.e*., the group containing the Western Hemisphere genera *Corystosiren*, *Culebratherium*, *Dioplotherium*, and *Xenosiren*—was long-lived, with a common ancestor estimated to be ~27.1 Ma (mid-Oligocene) and descendent fossil occurrences ranging from the terminal Oligocene to near the Miocene-Pliocene boundary. This monophyletic group is well-supported (PP = 0.87), but we detected no unambiguous synapomorphies along their joint stem. The placement of *Dioplotherium manigaulti* (Domning, 1989) as the basal-most member of this clade renders *Dioplotherium* paraphyletic, as younger *Dioplotherium* species are more closely related to other genera (Fig. 4). A dispersal out of the Gulf of Mexico region and into the Pacific, leading to occurrences of *Dioplotherium allisoni* in California and the Baja California Peninsula (Fig. 5), was estimated to have occurred between ~18.6 and ~16.6 Ma (Burdigalian, early Miocene), well before the closing of the Central American Seaway (O’Dea et al., 2016). A clade containing *Corystosiren* and *Xenosiren* is robust (PP = 0.92), with a common ancestor estimated to be ~11.7 Ma (terminal middle Miocene) and geographically reconstructed between the Yucatan and Florida Peninsulas. The *Corystosiren-Xenosiren* clade is supported by three unambiguous synapomorphies (10.1, palate >1 cm thick at level of penultimate tooth; 20.1, frontal roof bears bilateral pair of knoblike bosses, more or less cylindrical in shape and directed anterad, or at least a distinct longitudinal ridge of swelling medial and parallel to, and distinct from, each temporal crest; 60.3, cross-section of I^1^ crown broad and extremely flattened mediolaterally).

### Origin and evolution of crown Dugongidae

We acknowledge that crown groups are technically defined by extant taxa. However, in the case of the recently extinct species *Hydrodamalis gigas* (Steller’s sea cow), we note that the taxon is known from soft and hard tissues, abundant DNA evidence, and by scientific documentation of living animals as recently as the 18th century. Therefore, the phylogenetic impact of *H. gigas* in our study is essentially equivalent to that of an extant species. Accordingly, we have elected to define the crown Dugongidae clade by the last common ancestor of *Dugong dugon* and *H. gigas*. Previous studies have applied the same definition (*e.g*., Springer et al., 2015).

As previously noted, a highly credible *Dugong*-*Nanosiren* group was variably placed by the TE and M+B analyses (Fig. 4 and Fig. S1). This topological difference has implications for clade membership and the estimated diversification age of crown Dugongidae—and reflects the important role of molecular evolutionary rates in constraining the TE topology (see Discussion). Both analyses recovered the subfamilies Dugonginae and Hydrodamalinae as reciprocally monophyletic groups that are joined at a crown Dugongidae node. In the TE result, the crown dugongid split was estimated to be ~21.2 Ma (Aquitanian, earliest Miocene) and was reconstructed in the west Atlantic, slightly north of Florida.

Both analyses also found the genus *Metaxytherium* to be paraphyletic, which is consistent with previous assessments by other authors (*e.g*., Domning & Pervesler, 2012). In the TE result, two clades of *Metaxytherium* species successively branch from the stem lineage prior to the crown dugongid node. The more basal of these diversifications includes late Oligocene occurrences of *M. albifontanum* from the west Atlantic (Vélez-Juarbe & Domning, 2014a), and the more advanced split leads to a clade of four *Metaxytherium* species (*M. krahuletzi*, *M. medium*, *M. subapenninum*, and *M. serresii*) from the Eastern Hemisphere. The Europe-bound trans-Atlantic dispersal that led to this latter *Metaxytherium* group (Fig. 5) was estimated to have occurred between ~24 and ~21.4 Ma (near the Oligocene-Miocene boundary), and gave rise to a strongly supported (PP = 1.0) and long-lived (~18 million-year-long) clade that eventually went extinct in the Pliocene.

The middle and middle-to-late Miocene species *Metaxytherium crataegense* (Simpson, 1932) and *Metaxytherium floridanum* (Hay, 1922; Domning, 1988) were identified as stem dugongines, being placed as consecutive sister taxa of the *Dugong*-*Nanosiren* clade. The common ancestor of this latter group was estimated to be ~12.2 Ma (Serravallian, late middle Miocene) and was reconstructed near Florida. Originating from this area, the South China Sea-bound trans-Pacific dispersal that was modeled for the lineage leading to *Dugong dugon*, must have occurred sometime after the ~12.2 Ma split from *Nanosiren*, but presumably before the effective closure of the Central American Seaway at ~2.8 Ma (O’Dea et al., 2016). Seven unambiguous synapomorphies support the *Dugong* + *Nanosiren* association (7.0, zygomatic-orbital bridge of maxilla nearly level with palate; 9.1, zygomatic-orbital bridge of maxilla shortened, thickness greater than or equal to 0.40 × length; 18.1, frontal roof deeply concave or depressed overall, with or without a small median convexity between temporal crests, but not sloping ventrad anteriorly; 20.1, frontal roof bears bilateral pair of knoblike bosses, more or less cylindrical in shape and directed anterad, or at least a distinct longitudinal ridge or swelling medial and parallel to, and distinct from, each temporal crest; 25.1, dorsolateral border of exoccipital thicker, rounded, and more or less smooth, ca. 0.5–1.5 cm thick; 30.3, processus retroversus of the squamosal present, strongly inflected; 38.1, ventral rim of orbit does distinctly overhang the lateral surface of the jugal).

An entirely Pacific hydrodamaline clade was recovered with high credibility values at the subfamilial node and all descendent splits (PPs >= 0.98). This group includes *Hydrodamalis* species, *Dusisiren* species, and *Metaxytherium arctodites*, with a common ancestor estimated to be ~18.5 Ma (Burdigalian, late early Miocene) and geographically reconstructed near the northern portion of the Baja California Peninsula. From a west Atlantic origin, the Pacific-bound dispersal of the lineage leading to Hydrodamalinae was estimated to have occurred between ~21.2 and ~18.5 Ma (early Miocene; Fig. 5), an age that correlates with an open Central American Seaway (O’Dea et al., 2016). Monophyly of this subfamily is supported by one unambiguous synapomorphy (13.1, zygomatic-orbital bridge of maxilla, both edges thin and sharp). Biogeographic reconstruction placed most hydrodamaline lineages near California and the Baja California Peninsula. The group gave rise to the genus *Hydrodamalis* at ~7.9 Ma after which, but before ~5 Ma, one *Hydrodamalis* lineage dispersed into the northern Pacific (late Miocene to earliest Pliocene; Fig. 5) and split into *H. gigas* and *H. spissa*. The occurrence of *Dusisiren dewana* in Japan is phylogenetically constrained to represent an independent movement of hydrodamalines into the northern Pacific.

### Lineage diversity through time

**Figure 6:**
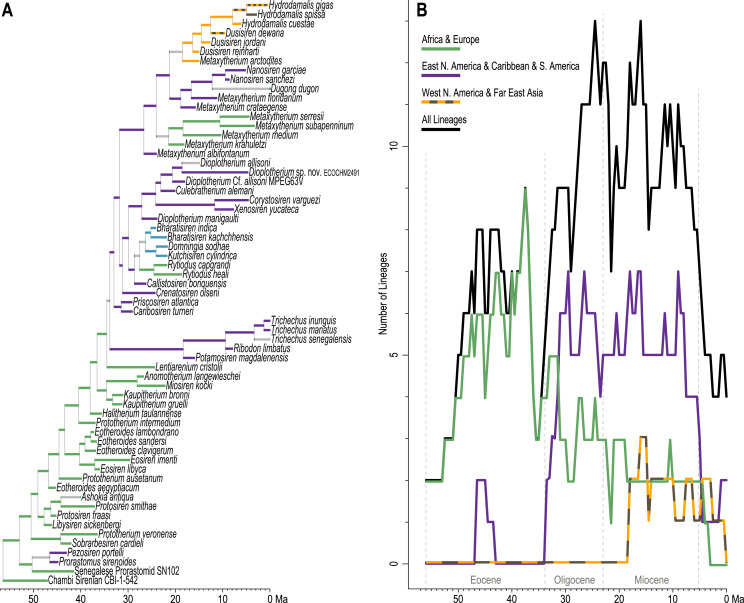
Lineages through time assessment. (A) Total evidence tree pruned to include only the latest occurrences of each sirenian species—branches are colored in correspondence to unambiguous reconstructions from Fig. 5B. (B) Number of lineages sampled in half-million-year increments, characterized for the full tree and by geographic zones.

Sirenian lineage diversity, quantified at half-million-year increments, is summarized in Fig. 6. As our phylogenetic analysis excludes many fossil species that are unrepresented in the morphological matrices of Furusawa (2004), Benoit et al. (2013), and Vélez-Juarbe & Wood (2018), this time-slice “lineages through time” (LTT) plot should be interpreted as minimum diversity estimates at all temporal samples. In addition to summarizing the full TE time-tree, we subsetted tree branches into their reconstructed geographic zones and quantified per-zone diversity at the same sample ages.

Diversity within the order steadily increased through the Eocene, to a peak of nine lineages at 37.5 Ma (near the Bartonian-Priabonian boundary). The subsetted geographic LTTs reveal that—other than a peak of two Caribbean prorastomid lineages quantified between 46.5 and 43.5 Ma—sirenian diversity during the Eocene was predominantly centered in the Tethys region of the Eastern Hemisphere (essentially northern Africa and southern Europe). A diversity crash occurred in that region during the latest Eocene, dropping to *only three* lineages just before the EOB. After a modest and short-lived diversity increase during the early Oligocene, African and European lineage counts steadily decreased through the Oligocene and Miocene. In stark contrast, the EOB marked the approximate onset of a rapid diversity increase in the geographic zone that includes the Caribbean, eastern North America, and South America. This region quickly reached seven lineages during the early Oligocene (sampled at 29.5 Ma), and continuously supported four to seven lineages until just before the Miocene-Pliocene boundary (sampled at 5.5 Ma).

Considering the full tree without geographic subsetting, sirenian diversity peaked at 13 lineages during the late Oligocene (sampled at 24.5 Ma), dropped in the earliest Miocene, and then recovered to 13 lineages near the Serravallian-Tortonian boundary (sampled at 16 Ma). This pattern reflects persistent diversity in the Caribbean and northern-adjacent west Atlantic, combined with the Pacific diversification of hydrodamalines (first sampled as a geographic subset at 18 Ma). Since the late Miocene, sirenian diversity has declined precipitously, from 11 lineages (sampled at 9 Ma) to the four remaining extant species of sea cows.

## Discussion

The total evidence phylogenetic assessment presented in this study is novel by integrating much of the morphological, molecular, temporal, and geographic data that inform the evolutionary relationships of sea cows. The accompanying model of ancestral biogeography is also new and uniquely combines continuous-trait geospatial data, a phylogenetic framework, and a broad temporal and taxonomic sample into an algorithm-derived hypothesis. These results have yielded a compelling new context for understanding the Earth history factors (*e.g*., oceanographic, climatic, geologic) that might have been associated with, or might have driven, adaptations and dispersal events that occurred in Sirenia’s evolutionary past.

Before discussing the inferences that can be drawn from our analyses, we raise two important caveats. First, the geographic breadth of the individual extant sirenian species (Fig. 1) cannot be represented by a single geospatial point—we acknowledge that the same surely holds for extinct sirenians. To the extent possible, we have included multiple occurrence localities for individual fossil species as a strategy to incorporate spatial variation, but we also recognize that the incomplete nature of the fossil record limits our ability to know, and sample from, their full geographic ranges. Analogously, our geographic reconstructions for lineage splits throughout sirenian phylogeny, include geocentroid points and associated geospatial density ellipses (*OSF Data Supplement*) as estimates for ancestral areas of occurrence. Therefore, the discussion that follows effectively treats these reconstructions as possible centers of larger geographic distributions. Second, many fossils that document sirenian evolution are simply too fragmentary to be of phylogenetic utility, and therefore were not included in our analyses. For example, Zalmout & Gingerich (2012) list several intriguing fossil occurrences from poorly sampled areas (see their Table 1). So, like most studies that incorporate paleontological evidence, our dataset is inexhaustive. Notwithstanding these qualifications, we consider the taxonomic, character, geospatial, and temporal sample included in the present study to be comprehensive enough to yield phylogenetic and biogeographic models that demonstrate broad evolutionary patterns and that will be robust despite additional sampling.

### Origin and early evolution of Pan-Sirenia

Although there seems to be a substantial gap in the order’s early fossil record, there is now broad consensus that Pan-Sirenia’s origins were probably associated with the Afro-Arabian landmass. The same biogeographic hypothesis can be stated for the other two extant paenungulate orders (Proboscidea and Hyracoidea). The first appearance of the extinct paenungulate order Embrithopoda is in the early Eocene of Morocco (Gheerbrant et al., 2021). The embrithopod group has been phylogenetically placed as the sister of Sirenia to the exclusion of Proboscidea (Seiffert, 2007; Cooper et al., 2014), or as the sister of a Sirenia + Proboscidea clade (Gheerbrant et al., 2021). In either case, the geographic signal from all the paenungulate orders seems to indicate that their individual and joint origins were Afro-Arabian, and proximate to the Tethys Sea. Paradoxically, it was until relatively recently that the earliest known occurrence of fossil sirenians was from the middle Eocene of Jamaica (*i.e*., *Prorastomus*). However, newer discoveries of *Libysiren* from Libya (Domning, Heal & Sorbi, 2017) and the primitive petrosal CBI-1-542 from Chambi, Tunisia (Benoit et al., 2013) can now be considered to predate the Jamaican fossils. These data provide compelling evidence for the presence of Pan-Sirenia in Afro-Arabia near the early-middle Eocene boundary (Benoit et al., 2013). A fragmentary prorastomid vertebra from the middle Eocene of Senegal (Hautier et al., 2012) is another African specimen that is of outsized taxonomic and biogeographic importance.

Our analyses strongly support the phylogenetic placement of the Tunisian CBI-1-542 petrosal as the oldest and most basal stem sea cow. Further, our model reconstructed the biogeographic origin of Pan-Sirenia in northern Africa near the end of the late Paleocene (~56.4 Ma). More specifically, this analysis identified a region in the western portion of the Tethys Sea (Fig. 5) as both the center of evolution for the sirenian stem lineage, and the point of departure for the prorastomid dispersal to the Caribbean which was estimated to have occurred between ~50.3 and ~46.5 Ma. Given these results, we consider an alternative scenario of the sirenian stem lineage passing through the Caribbean and into the west Atlantic (Benoit et al., 2013, their Fig. 6) to be less likely.

The earliest trans-Atlantic dispersal of cetaceans probably occurred between ~45.7 and ~43.3 Ma (Gohar et al., 2021) and gave rise to the archaeocetes *Peregocetus* from Peru (Lambert et al., 2019) and *Carolinacetus* from North America (Geisler, Sanders & Luo, 2009). This temporal window does not overlap with our estimate for the dispersal of prorastomids from the Tethys to the Caribbean. Therefore, based on available evidence, we infer that sirenians were the first marine mammals of the Western Hemisphere and that the initial trans-Atlantic dispersals of sirenians and cetaceans were not correlated.

Our study does not include the poorly known Bartonian + Priabonian (41-33.9 Ma) fossil sea cows of the eastern United States and Mexico (Domning, Morgan & Ray, 1982) that could be the descendants of a second Eocene sirenian dispersal out of the Eastern Hemisphere. Domning (2001b) argued that these North American collections include prorastomids and protosirenids. Prorastomids could be late-surviving endemics related to *Prorastomus* and *Pezosiren*. While Beatty & Geisler (2010) diagnosed a Bartonian specimen from South Carolina as *Protosiren* sp., Zalmout & Gingerich (2012) argued that the specimens identified by Domning, Morgan & Ray (1982) as protosirenids are members of the genus *Eotheroides*. So, at present, there is not a consensus concerning some of these taxonomic referrals. Nevertheless, the *Eotheroides* clade recovered by our analyses first appeared in the Bartonian Tethys and the *Protosiren* + *Ashokia* clade arose in the Lutetian Tethys and survived into the Bartonian. Therefore, these occurrences of *Protosiren* and/or *Eotheroides* in North America signal a trans-Atlantic dispersal that might have been broadly contemporaneous with the Bartonian-aged (Gohar et al., 2021) dispersal of basilosaurid cetaceans such as *Basilosaurus* and *Dorudon*, which are similarly known from both northern Egypt and the eastern United States (*e.g*., Gingerich, 1982; Smith et al., 2022).

### Origin of crown Sirenia at the Eocene-Oligocene boundary

Notable among our results is the novel detection of a terminal Eocene dispersal (~34.5-33.9 Ma) for advanced stem sirenians from Europe to the west Atlantic, giving rise to crown Sirenia effectively at the Eocene-Oligocene boundary (33.9 Ma). The age of this event is associated with one of the most dramatic climatic shifts of the entire Cenozoic, involving rapid and widespread glaciation of Antarctica, a major drop in global sea levels, lowering of the calcite compensation depth, increased seasonality and cooler winter temperatures at northern latitudes, and the onset or strengthening of the Atlantic meridional overturning circulation (*e.g*., Abelson & Erez, 2017; Coxall et al., 2005; Eldrett et al., 2009; Hutchinson et al., 2019, 2021; Miller et al., 2020).

EOB-related extinctions were particularly severe in the marine realm (*e.g*., Raup & Sepkoski, 1986), which accords with our LTT assessment (Fig. 6) that detected a major loss of sirenian diversity near the terminal Eocene, leading to the lowest numbers in the entire Cenozoic, including the present—*i.e*., down to *only three* lineages. These extinctions were presumably tied both to cooling and to the rapid drop in sea levels that occurred at this time, as most living and extinct sirenians are assumed to have been dependent on seagrasses growing in relatively shallow and warm waters, and nearshore seagrass communities were presumably significantly perturbed by these climatic and eustatic events. However, our analyses also suggest that the trans-Atlantic dispersal to subtropical (or tropical) latitudes at the EOB led to a diversity rebound, with an increase to seven Western Hemisphere lineages within the first five million years of the Oligocene. Sirenian diversity continued to increase through the Oligocene, reaching peak levels around 24.5 Ma. We note that the two diversity troughs detected for African and European sirenian lineages (Fig. 6)—near the EOB and during the early Oligocene—are similar to the pattern presented by de Vries et al. (2021) for African terrestrial mammals.

An estimated age of ~33.9 Ma for the Trichechidae-Dugongidae split (this study) stands in stark contrast to the ~41.6 Ma (latest Lutetian) hypothesis of Springer et al. (2015), whose parsimony analysis of morphological data led them to select *Eotheroides aegyptiacum* from the middle Eocene as a fossil calibrator for crown Sirenia. BTD analyses instead placed *E. aegyptiacum* as one of the most basal stem sirenians, only one split more advanced than Protosirenidae. Even the later occurring *Eotheroides* species, herein placed more crownward than *E. aegyptiacum*, retained femora and relatively well-developed innominate bones (Zalmout & Gingerich, 2012) that would—if placed within the crown group—imply that hindlimb loss occurred independently in dugongs and manatees, which only retain pelvic vestiges (Domning, 1991; Fagone, Rommel & Bolen, 2000; Nganvongpanit et al., 2020). The exclusion of *Eotheroides* from crown Sirenia allows for a last common ancestor of extant sea cows that would have lacked hindlimbs entirely.

The more recent parsimony analysis of Vélez-Juarbe & Wood (2018) excluded all *Eotheroides* species from crown Sirenia. This hypothesis, which was inferred from one of the character matrices included in our morphological data partition, is more consistent with the BTD results presented here. When comparing the Vélez-Juarbe & Wood (2018) tree to the results of our study, the only differences in the taxonomic content of crown Sirenia are *Kaupitherium* (early Oligocene of Europe), *Lentiarenium* (late Oligocene of Europe), and the miosirenines (European *Anomotherium* and *Miosiren* from the late Oligocene and early Miocene, respectively). Total evidence BTD analysis placed these taxa as the most advanced stem sirenians, whereas parsimony methods have placed them as nested within the crown group. The parsimony topology implies a radically different biogeographic scenario where the last common ancestor of Trichechidae and Dugongidae was located in the Eastern Hemisphere (rather than in the west Atlantic) and, after the crown Sirenia split, the antecedents of each of these lineages independently dispersed across the Atlantic Ocean. However, the parsimony result does not necessarily imply an older age for crown Sirenia, as its oldest crown clade taxon (*Kaupitherium*, early Oligocene) postdates the EOB.

The total evidence BTD placement of miosirenines as stem sirenians, rather than as stem trichechids, is unique to this study. The morphological character states that have been highlighted as common to both Miosireninae (*i.e*., *Anomotherium* + *Miosiren*) and Trichechinae (*i.e*., *Potamosiren* + *Ribodon* + *Trichechus*), and which could affiliate miosirenines with the trichechid stem lineage, include: a rostrum that is small relative to the cranium (Domning, 1994; character 3, state 0); absence of the processus retroversus (Domning, 1994; character 77, state 0); and an external auditory meatus that is wider than it is tall (Domning, 1994; character 82, state 2). In the character-taxon matrices of Domning (1994) and Vélez-Juarbe & Wood (2018), these traits are unscored for *Potamosiren* and *Ribodon* because the available fossil material does not preserve the relevant anatomy. Therefore, empirically shared states are between miosirenines (>~22 Ma) and extant *Trichechus* species. In the later matrix (which was used in our study), the “small rostrum” state is shared by miosirenines, *Trichechus*, and several stem sirenians (*e.g*., *Prorastomus*, *Pezosiren*, *Protosiren*, *Libysiren*, *Eotheroides*, and *Prototherium*); an “absent processus retroversus” is shared by miosirenines, *Trichechus*, prorastomids, and protosirenids; and a “relatively wide external auditory meatus” is shared only by miosirenines, *Trichechus*, and *Ashokia* (see ASR of morphological character states 1.1, 30.0, and 32.2). We found the parsimony analysis of Vélez-Juarbe & Wood (2018) to be perfectly replicable (*OSF Data Supplement*) and added bootstrap resampling to their settings to produce a support-metric for the placement of miosirenines as stem trichechids. That re-analysis yielded a bootstrap proportion of only 24% for the Miosireninae + Trichechinae node. Our total evidence BTD assessment differs from the parsimony paradigm by evaluating morphological character states within a probabilistic framework, by incorporating the geologic ages of (and morphological distances among) sampled taxa, and by coding longitudinal zones as a biogeographic character. These factors have evidently overruled the relatively weak morphological evidence that could signal a direct affiliation of miosirenines with trichechids. In our TE time-tree, the two nodes that exclude miosirenines from the crown Sirenia clade (*i.e*., crown Sirenia and crown Sirenia + *Lentiarenium*) are together supported by five unambiguous synapomorphies (reported above). Four of these character states are not present in miosirenines (see ASR of morphological character states 15.1, 50.3, 64.2, and 70.2). To statistically test these competing topological hypotheses, we first generated an alternate tree by re-analyzing the TE supermatrix with a constraint-set that forced miosirenines to the trichechid stem, allowed node and tip ages associated with fossil trichechids and miosirenines to be re-estimated, but otherwise replicated the TE time-tree. We then used stepping-stone sampled optimization of the TE supermatrix against this alternate tree (*OSF Data Supplement*) to generate a marginal log-likelihood value for the “miosirenines as stem trichechids” hypothesis and compared this metric to the previously reported value for our optimal TE time-tree. The optimal hypothesis (marginal LnL = −215136.32) outperformed the alternate hypothesis (marginal LnL = −215140.24) with Ln(BayesFactor) = 3.92 (Basu & Chib, 2003). We interpret this value as very strong evidence (*i.e*., odds >50:1) (Jarosz & Wiley, 2014) in favor of a stem sirenian position for miosirenines. If the parsimony-based systematic hypothesis is rejected, it brings into question the validity of the subfamily nomen “Miosireninae” because it is potentially orphaned outside of any designated taxonomic family.

Additionally, we note that the total evidence BTD analysis yielded an older age estimate for crown Sirenia (~33.9 Ma; Fig. 4) than did the fossil-calibrated clock analysis of DNA alone (~30.6 Ma; Fig. 3). This despite both assessments being critically informed by *Priscosiren* as the oldest crown sirenian (either algorithmically placed or *a priori* assigned). This reflects the fact that, when faced with a highly incomplete fossil record, the oldest fossil attributable to a crown clade is unlikely to coincide with the divergence age of interest. In other words, to be identifiable as a fossil calibrator, an extinct taxon must already possess the anatomical apomorphies that allow it to be placed within a crown group. In the case of our DNA clock analysis, the chosen fossil calibrator (*Priscosiren*) possesses probable dugongid apomorphies, but the only available method for estimating the time it took to accumulate those traits is the use of a “morphological clock”, as is uniquely possible in BTD analyses (*e.g*., Ronquist et al., 2012a). In our total evidence BTD result, the morphological distance implied by the distribution of character states requires a crown split that is ~4.4 million years older than the oldest crown species. The younger age estimated by the DNA clock analysis can be (at least partly) attributed to the absence of any information concerning rates of morphological evolution.

### Origin and evolution of trichechids

Despite an origin of the trichechid stem lineage at the Eocene-Oligocene boundary, the oldest known fossil attributable to this group is from the early Miocene of Peru (~21-20.1 Ma, SALMA-Colhuehuapian) (Antoine et al., 2016; Dunn et al., 2013). Due to its fragmentary nature, our morphological dataset did not include this specimen. Nevertheless, the TE analysis yielded a median age estimate of ~18.3 Ma (23.6-15.8 Ma HDI) for the ancestral node of the clade containing middle Miocene *Potamosiren*, late Miocene *Ribodon*, and extant *Trichechus* (*i.e*., Pan-Trichechidae, or Trichechinae *sensu* Domning, 1994). Even if we assume that the Peruvian trichechid slightly predates our Pan-Trichechidae nodal age estimate, and perhaps diverged from the stem prior to this node, the scenario still requires a trichechid ghost lineage of at least 12.9 million years through the entire Oligocene and part of the early Miocene. With biogeographic reconstructions for crown Sirenia near the Bahamas (~33.9 Ma) and Pan-Trichechidae in Colombia (~18.3 Ma), our analyses predict that stem trichechids would have been present in the Caribbean Basin at some point during the Oligocene, perhaps along the northern coast of South America.

**Figure 7:**
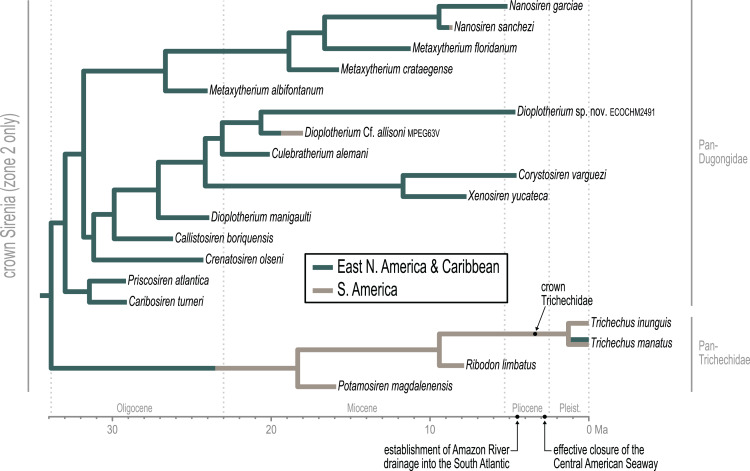
Phylogeny of crown sirenian species reconstructed in geographic zone 2. Tree is from Fig. 6A and was modified by pruning non-crown taxa and all taxa reconstructed in other zones. Branch colors correspond to the geocoordinates-based reconstruction presented in Fig. 5A.

Further, our model suggests that from the early Miocene to (at least) the late Pliocene, trichechids were entirely restricted to mainland South America (Figs. 5 and 7). The ~18.3 Ma node that links *Potamosiren* to all later trichechids was reconstructed in central Colombia, while the ~9.4 Ma ancestor of *Ribodon* and *Trichechus* was estimated near the joint borders of Peru, Brazil, and Bolivia. Divergences among the extant *Trichechus* species were reconstructed in northwestern Brazil, near the modern-day Rio Negro, with the crown Trichechidae node estimated to be ~3.3 Ma and the *T. manatus-T. inunguis* split to be ~1.3 Ma. de Souza et al. (2021) estimated a substantially older age for crown Trichechidae (~6.56 Ma), but their use of the undoubted stem sirenian *Prorastomus* as a fossil calibration for crown Sirenia likely led to unrealistically old divergence times.

Occurrences of the oldest fossil trichechids and their reconstructed ancestors in the early Miocene of northwestern South America (Peru and Colombia), and subsequent late Miocene splits reconstructed within Brazil, can be explained by the ancient presence of an extensive inland area of freshwater mega-wetlands known as the Pebas System. The Pebas covered much of western Amazonia from ~20.4 to ~4.5 Ma, reached its peak areal extent in the middle Miocene, and connected to the proto-Caribbean (*via* modern-day Colombia and Venezuela) for much of the early and middle Miocene (Albert, Val & Hoorn, 2018; Hoorn et al., 2022; de Souza et al., 2021; Suarez et al., 2021). We propose that even the late Miocene occurrence of *Ribodon* in Argentina might be explained by an ancient freshwater connection with the Pebas System, as has recently been suggested for freshwater potamotrygonine stingrays, the phylogeny and distribution of which suggest a connection between the Upper Amazon and the Parana-Paraguay regions during the late Miocene (Fontenelle et al., 2021). During South America’s mega-wetland evolution, communication was established between the Upper and Lower Amazon by ~10 Ma, and large-scale eastward flow of the Amazon toward the South Atlantic began ~4.5 Ma (Albert, Val & Hoorn, 2018). We infer that the formation of these connections might have allowed near-crown trichechids to colonize first the Acre mega-wetlands from the diminishing Pebas System (~10 Ma), and then later the transcontinental Amazon. This paleobiogeographic hypothesis for trichechid evolution resembles both the “alternative scenario” proposed by Domning (1982) and the more recent model of de Souza et al. (2021).

There is no unambiguous fossil evidence of *Trichechus* outside of mainland South America until the Pleistocene, with the oldest specimens that have been recovered *in situ* (*i.e*., the Leisey sample from the NALMA-Irvingtonian of Florida) likely 1.3-1.1 Ma (Domning, 2005). Our analyses revealed no temporal overlap between the estimated age of the crown Trichechidae node (~3.3 Ma) and the tip ages of the latest occurring North American rytiodontine dugongids (*i.e*., *Corystosiren varguezi* and *Dioplotherium* sp. nov., ~4.5-4.6 Ma; Fig. 7). Given the intra-Amazonian origin of *Trichechus* suggested by our biogeographic reconstruction, these results provide a bound of ~3.3 Ma for the earliest possible movement of crown *Trichechus* out of South America. The beginning of Amazon discharge into the South Atlantic at ~4.5 Ma (Albert, Val & Hoorn, 2018) only slightly predates (and is within the HDI range of) the crown Trichechidae split. We infer that the establishment of this drainage pattern made possible the movement of *Trichechus* into the South Atlantic only shortly thereafter. The ~3.3 Ma crown Trichechidae estimate is also the earliest possible age for the trans-Atlantic dispersal of the *T. senegalensis* lineage from South America to western Africa. Considering the estimated rate of seafloor spreading (Pérez-Díaz & Eagles, 2014), the relatively young temporal window for this dispersal indicates a trans-Atlantic distance that was only moderately shorter than modern. More narrowly, the age of the spread of *T. manatus* beyond South America is constrained to sometime after the *T. manatus*-*T. inunguis* split (~1.3 Ma, mid-Pleistocene; Fig. 7). The estimated age of this divergence curiously correlates with the maximum probable age of the earliest known *T. manatus* fossils (*i.e*., the Leisey sample discussed above). Our analyses suggest that there was no temporospatial overlap of dugongids and *Trichechus*, even when an undescribed *Dugong*-like dugongine from the late Pliocene of Florida is taken into account (Domning, 2001b). At present, there is no basis to suspect that trichechids and dugongids simultaneously occurred in the Caribbean or northern-adjacent west Atlantic during the Plio-Pleistocene.

We note that Domning (2001b) proposed that a maxillary specimen (USNM 167655) from North Carolina, that has been attributed to *Ribodon* but is of unknown age and provenance, is “possibly of correlative age” to the late Miocene *Ribodon* specimens from Brazil and Argentina. More recently, Suarez et al. (2021) suggested that the specimen is “presumably from Pliocene deposits”. We do not consider this specimen to provide reliable direct-evidence for a pre-Piacenzian (or pre-Pleistocene) occurrence of trichechids outside of South America.

### Evolution of persistent dugongid diversity in the Caribbean and west Atlantic

Our analyses indicate that, from the earliest Oligocene to the Pliocene, the evolution of Pan-Dugongidae has been centered in the Caribbean and northern-adjacent west Atlantic, and that this region served as the point of departure for: (a) two back-dispersals into the Eastern Hemisphere (first a rytiodontine clade in the late Oligocene, and later a *Metaxytherium* lineage in the latest Oligocene or early Miocene); (b) two early Miocene dispersals into the Pacific Ocean (hydrodamalines and the *Dioplotherium allisoni* lineage); and (c) one later Miocene or Pliocene dispersal across the Pacific into what is now the South China Sea [*Dugong*]. The TE time-tree requires that, from ~31.5 to ~5.5 Ma, no fewer than three, and as many as six, dugongid lineages simultaneously inhabited this region (Fig. 7; zone 2 excluding South America). Although not all of these sea cows were necessarily sympatric, the presence of such high (and sustained) dugongid diversity in a restricted region is nevertheless perplexing—given the specialized and canalized nature of the ancestral crown sirenian bauplan, which presumably restricts morphological evolvability. Sirenians also have access to far less plant diversity in their aquatic habitats than do terrestrial herbivores (Domning, 1978).

Domning (2001b) and Vélez-Juarbe, Domning & Pyenson (2012) suggested that multispecies assemblages of dugongids might have been accommodated by divergent evolution of ecomorphological features such as body mass, rostral deflection, and tusk size and morphology in sympatric species. Our analyses indicate that rates of morphological evolution (*OSF Data Supplement*) during the early Oligocene, characterized at the Rytiodontinae node and at that clade’s earliest non-*Crenatosiren* split were higher, or far higher, than nearly all other splits in the Pan-Dugongidae group. This pattern is consistent with a scenario in which early rytiodontines were rapidly evolving to exploit newly available niches. Between ~31.2 and ~29.9 Ma (early Oligocene), rytiodontines evolved relatively large tusks with a lozenge-shaped cross section (see ASR of morphological character states 59.2 and 60.2). These larger tusks have been functionally linked to the excavation of relatively large seagrass rhizomes, a resource that likely would have been unavailable to other stem dugongids (Domning, 2001b; Vélez-Juarbe, Domning & Pyenson, 2012). Later, between ~29.9 and ~27.1 Ma, a specialized “butt joint” between the premaxilla and frontal (see ASR of morphological character state 2.2) evolved along the lineage leading to the rytiodontine clade that contains *Culebratherium*, *Corystosiren*, *Dioplotherium*, and *Xenosiren*. This trait has been interpreted as a mechanism to dissipate forces incurred when extracting large and deeply buried seagrass rhizomes (Domning, 2001b; Domning & Beatty, 2007). Large-tusked sirenians have been proposed as possible keystone members of these multispecies assemblages that could have prevented seagrass communities from being dominated by relatively large seagrass taxa—*i.e*., staving off a so-called “climax” state in which smaller seagrasses are excluded (Domning, 2001b; Vélez-Juarbe, Domning & Pyenson, 2012). Our analyses imply that relatively large-tusked rytiodontines with premaxilla-frontal “butt joints” were present in the Caribbean and northern-adjacent west Atlantic from the mid-Oligocene all the way into the early Pliocene (see the *Culebratherium* + *Corystosiren* + *Dioplotherium* + *Xenosiren* clade in Fig. 7), and might have played an important role in helping to maintain diverse seagrass communities that could, in turn, sustain diverse sirenian communities. Possible support for the interpretation of these sea cows as keystone members of long-lived multispecies communities comes from the fact that their disappearance was soon followed by the local extinction of all other dugongids in the region. Alternatively, the extirpation of dugongids in the west Atlantic might reflect a collapse of seagrass communities caused by paleoceanographic changes (Domning, 2001b) that were associated with the gradual constriction and final closure of the Central American Seaway during the Pliocene (O’Dea et al., 2016).

### Return to the Eastern Hemisphere: Eastward trans-Atlantic dugongid dispersals

Our analyses estimated an age between ~28.7 and ~27.6 Ma (terminal early Oligocene) for the first eastward trans-Atlantic dispersal of dugongids. Reconstructed as having moved into the Tethys from the west Atlantic (north of modern-day Cuba), this rytiodontine lineage gave rise to the relatively widespread form *Rytiodus* (known from southern Europe, northern Africa, and Madagascar) and the South Asian genera *Bharatisiren*, *Domningia*, and *Kutchisiren*. This age interval predates a late Oligocene phase of sea surface warming (“late Oligocene warming” or LOW, ~26.5-24 Ma) when both tropical and global sea surface temperatures returned to late Eocene levels (O’Brien et al., 2020). While our results do not suggest that this dispersal and the LOW phase were temporally linked, the warming event likely impacted the environmental conditions of the late Oligocene Tethys in a manner favorable to rytiodontines after they arrived. Further, the TE phylogenetic analysis placed the four late Oligocene rytiodontine species from South Asia in an exclusive group—and the lineage leading to this clade was reconstructed as a single dispersal (~27.6-26.3 Ma) from the eastern Tethys into the Indian Ocean. Three of these species occurred contemporaneously and, given their close geographic proximity, were likely sympatric. Additionally, they differed in body size and degree of rostral deflection which have been interpreted as ecomorphological traits tied to variable foraging preferences (Vélez-Juarbe, Domning & Pyenson, 2012). A monophyletic origin of this multispecies sympatric group requires that the three ecomorphs evolved during an interval of ~4.6 million years (~26.3-21.7 Ma) from a common ancestor. The occurrence of *Rytiodus heali* in Madagascar was reconstructed as a second rytiodontine dispersal from the eastern Tethys into the Indian Ocean—an event that is constrained to sometime after the taxon’s preceding lineage split (~24.5 Ma), but presumably before the effective closure of the Indian Ocean-Mediterranean Seaway (~21-20 Ma, Torfstein & Steinberg, 2020).

A second dugongid trans-Atlantic dispersal out of the Western Hemisphere (in this case towards Europe) was reconstructed as having occurred between ~24 and ~21.4 Ma (terminal Oligocene to earliest Miocene). This dispersing lineage gave rise to several European (and at least one North African) occurrences of *Metaxytherium* species. The event postdates the LOW, but is possibly correlative with a later phase of sea surface warming during the earliest Miocene (~22.3 Ma; Guitián & Stoll, 2021). Our TE time-tree suggests that there was potential temporospatial overlap in southern Europe and the Mediterranean of this *Metaxytherium* clade with the rytiodontine *Rytiodus* (which disappeared in the late early Miocene)—a scenario that accords with previously published interpretations (*e.g*., Domning & Sorbi, 2011). However, the sirenian faunas of Europe and Africa throughout the entire Miocene were relatively depauperate compared to contemporaneous communities in the Caribbean and northern-adjacent west Atlantic (Fig. 6). *Metaxytherium* persisted in the Mediterranean until the late Pliocene (Sorbi et al., 2012). Several environmental factors are likely to have contributed to the decreasing diversity and ultimate extinction of these Mediterranean sea cows, notably including degradation of habitats during the Messinian Salinity Crisis (*e.g*., Carone & Domning, 2007; Sorbi et al., 2012). The final disappearance of *Metaxytherium* near the end of the Pliocene is, however, temporally coincident with a major extinction event that independently impacted several lineages of marine megafauna (Pimiento et al., 2017). This diversity crash might have been driven by a substantial drop in sea levels (Dumitru et al., 2021) and an associated loss of productive habitats in the neritic zone (Pimiento et al., 2017). This pattern potentially mirrors the post-EOB extinctions that were concomitant with falling sea levels.

### Colonization of the Pacific Ocean and the origin of *Dugong*

Domning & Furusawa (1994) argued that “sirenians undoubtedly occurred (in the North Pacific) in the Eocene and Oligocene, but we know nothing of their history or diversity. Therefore, we are on shaky ground when we interpret the Neogene record as though the forms that appear after the start of the Miocene had just dispersed through the Central American Seaway into an ocean devoid of sirenians.” While we acknowledge that sea cows might have been present, and possibly even taxonomically diverse, in parts of the Pacific during the Eocene or Oligocene, our analyses do not require a sirenian presence in the eastern Pacific until the early Miocene. One report of a fossil dugongid (gen. et sp. indet.) from Oregon is presumably earliest Miocene in age (23-22.5 Ma; Domning & Ray, 1986; Prothero et al., 2001; Raffi et al., 2020). This material is the earliest record of sirenians in the eastern Pacific but is unscored for phylogenetic analysis and was not included in the present study. Our phylogenetic hypothesis can temporally accommodate this Neogene occurrence within crown Sirenia but, if it is derived from that clade, the specimens’ geographic provenance does not require a dispersal of crown sea cows into the eastern Pacific earlier than the confidence intervals of our model. Additionally, two reports of Oligocene sea cows from the western Pacific (Japan) consist of sparse material that restricts phylogenetic assessment (Mori, Miyata & Kato, 2019; Okazaki, 1984). We cannot assume that these occurrences are derived from a crown sirenian lineage that dispersed westward from the Caribbean or from a stem sirenian lineage that dispersed eastward from the Indo-Pacific. We further note that it is not the case that there are no Paleogene marine deposits in which eastern Pacific sirenians could be preserved. For instance, the highly productive middle-to-late Eocene deposits of the Pisco Basin in Peru have not yet yielded remains of sirenians, whereas cetaceans and other marine megafauna are well-documented (Lambert et al., 2019; Malinverno et al., 2021). Similarly, the late Oligocene El Cien and San Gregorio formations of Baja California Sur have produced abundant cetaceans and elasmobranchs (among other marine vertebrates), but sea cows are absent from these assemblages thus far (Hernández Cisneros, González Barba & Fordyce, 2017). The Baja sites are notable not only for their geographic proximity to the Caribbean (which presumably would have been the source region for Pacific crown sirenians), or because the peninsula preserves some of the earliest known sea cows of the eastern Pacific (*i.e*., middle Miocene *Metaxytherium arctoides* and *Dusisiren reinharti*—but see the Oregon occurrence discussed above), but also that the El Cien sites represent neritic zone depths and paleoenvironments that include estuaries, deltas, shelf, and coastal lagoons (Schwennicke, 1994). These habitats are akin to those of the sirenian-bearing middle Miocene sites that are less than 200 km away. Additionally, if undetected Paleogene sea cows from the eastern Pacific were descendants of the crown Sirenia group (*i.e*., not descended from Caribbean prorastomids or North American *Protosiren* and/or *Eotheroides*) then, given our divergence age estimates and biogeographic model, the earliest possible age for a westward dispersal into the Pacific from the Caribbean or northern-adjacent west Atlantic would be sometime after the crown Sirenia split (~33.9 Ma). In this scenario, an eastern Pacific presence of sirenians during the Eocene could be ruled out.

Our results suggest new constraints for the timing of two early Miocene sirenian dispersals into the Pacific (Fig. 5). First, the dispersal of the ancestor of Hydrodamalinae (including *Metaxytherium arctodites*) was estimated to have occurred between ~21.2 and ~18.5 Ma, and second, the lineage that gave rise to *Dioplotherium allisoni* (with occurrences in southern California and Baja California Sur) between ~18.6 and ~16.6 Ma. The parent nodes of these lineages were reconstructed in Florida and the Gulf of Mexico, respectively, leaving no reason to suspect that either clade might be traced back to a more ancient Pacific ancestor. In light of our results, if there were any Paleogene sirenians in the eastern Pacific, they are unlikely to have had any relevance to the origin of Hydrodamalinae which is the major clade that would come to inhabit that ocean through most of the Neogene. Our analyses indicate that *Dusisiren dewana* was ranging into the northern Pacific by the middle Miocene (~11.4-9.7 Ma), and that the lineage leading to the *Hydrodamalis gigas* + *Hydrodamalis spissa* clade moved into the northern Pacific during the late Miocene or very early Pliocene (~7.9-5 Ma) with a last common ancestor of these two species reconstructed near Hokkaido. Domning (1978) provided a detailed synthesis of the Miocene-to-Recent evolution of hydrodamalines in the Pacific Ocean, none of which is inconsistent with the results presented here.

The origin of *Dugong*’s current Indo-Pacific distribution is one of the great mysteries of sirenian evolution. It remains difficult to reconcile with the sirenian fossil record, which strongly signals the Caribbean and northern-adjacent west Atlantic as having been the center of dugongid diversification since the early Oligocene, with an undescribed Floridian *Dugong*-like form persisting even into the late Pliocene (Domning, 2001b). To further complicate matters, the primary difference between the results of our BTD analyses (that include or exclude molecular data) is the phylogenetic position of *Dugong*. When molecular data were not accounted for (Fig. S1), the *Dugong* + *Nanosiren* clade was placed as the sister of all other crown dugongids (*i.e*., genera *Dusisiren*, *Hydrodamalis*, and *Metaxytherium*) with a divergence having occurred in the late Oligocene (~27.4 Ma). When molecular data were incorporated (Fig. 4), the *Dugong* + *Nanosiren* group was placed as nested within a clade that includes west Atlantic *Metaxytherium* species—and the last common lineage that led to this group was estimated to have diverged from the hydrodamaline group (*sensu* this study) in the early Miocene (~21.2 Ma). These contrasting results reflect the fact that a Paleogene split of the *Dugong* + *Nanosiren* clade from all other crown dugongids simply cannot be accommodated by the rates of molecular evolution required by our DNA supermatrix and numerous afrotherian fossil calibrations. Indeed, molecular data alone suggest an even younger (~13.3 Ma, middle Miocene; Fig. 3) origin of crown Dugongidae than the ~21.2 Ma split recovered by our total evidence BTD analysis. This is another example (as with crown Sirenia) where the explicit inclusion of age constrained fossil taxa and their morphology in an analysis that simultaneously assesses genetic data has yielded older age estimates for crown nodes than has a fossil-calibrated clock analysis of molecular data alone. The more recent (early and middle Miocene) estimates for crown Dugongidae required by the genetic sequences in both our TE and DNA-clock analyses—considered in tandem with the first appearance of rytiodontines (*i.e*., *Crenatosiren*) in the early Oligocene—temporally exclude the possibility that Rytiodontinae is a nested descendent of the *Dugong-Hydrodamalis* split, as has been proposed by parsimony-based studies (*e.g*., Domning, 1994; Vélez-Juarbe, Domning & Pyenson, 2012; Vélez-Juarbe & Wood, 2018). Likewise, a phylogenetic placement of *Dugong* among rytiodontine taxa is incompatible with molecular rates.

Our models suggest that the lineage leading to *Dugong dugon* (following its divergence from *Nanosiren*) dispersed into the Pacific from near Florida sometime between ~12.2 Ma and the present. We argue that the minimum bound of this dispersal should be ~2.8 Ma, which corresponds to the effective closure of the Central American Seaway (O’Dea et al., 2016). A trans-Pacific dispersal towards the South China Sea, rather than a trans-Atlantic dispersal around the Cape of Good Hope towards Africa’s East Coast, is further supported by phylogeographic analyses which indicate that the earliest splits among populations of extant *Dugong* are likely to have occurred in the Indo-Australian region rather than in the western Indian Ocean (Plön et al., 2019). A trans-Pacific route also would have been supported by (or even facilitated by) wind-driven surface currents and could have allowed the ancestors of extant *Dugong* to remain in warm tropical (or subtropical) waters, whereas the opposite would be true for a journey around the Cape of Good Hope. However, both scenarios require long-distance open-ocean voyages that were far out of the neritic zone and therefore devoid of rooted seagrasses, leaving it unclear how dugongs could have sustained the energy required for such an extensive journey. Nevertheless, our results demonstrate that such open-ocean dispersals must have occurred several times during the evolution of sirenians—including the relatively recent dispersal of the *Trichechus senegalensis* lineage across the Atlantic.

An intriguing piece of evidence that might be relevant to the ancestral biogeography of the *Dugong* lineage is the very fragmentary fossil remains (four vertebrae and three incomplete ribs) of a remarkably small sirenian from Papua New Guinea (PNG) (Fitzgerald, Vélez-Juarbe & Wells, 2013). The specimens are of Miocene age (>11.8 Ma, mid-Burdigalian to terminal Serravallian) and occur within the modern geographic distribution of *Dugong dugon* but are at present unattributed to any sirenian taxon below the ordinal rank. The vertebral epiphyses are unossified to the centra which might indicate a juvenile individual. However, unossified epiphyses are also the observed condition in extant *Dugong* adults. Therefore, it’s uncertain whether size can be explained by juvenility or by normal adulthood of a diminutive taxon. The only Neogene sea cow species that is comparably small to the PNG remains is *Nanosiren garciae* from the early Pliocene of Florida (Fitzgerald, Vélez-Juarbe & Wells, 2013). Notably, our TE analysis placed *Nanosiren* and *Dugong* as sister taxa. This phylogenetic connection implies a smaller-than-dugong ancestral body size for the common ancestor of the clade. A potentially testable hypothesis that emerges from this evidence is that the PNG fossils represent a small species that is a close relative of *Dugong dugon* to exclusion of *Nanosiren*. If this were the case, the age and geographic provenance of the PNG sirenian would support a possible late middle Miocene trans-Pacific dispersal of the *Dugong* lineage. Our estimated age for the *Dugong*-*Nanosiren* split (12.2 Ma, 9.2-16.2 Ma HDI) can accommodate this hypothesis as long as the PNG sirenian is younger than ~16 Ma. Nevertheless, there is direct fossil evidence of dugongines in southern Australia by the early Pliocene (Pledge, 2006). We propose that the presence of sirenians in the Australasian realm after the early Miocene is the result of a single trans-Pacific dispersal of the *Dugong* lineage. This biogeographic scenario requires no additional extraordinary trans-oceanic dispersals.

### Genetically detectable lineages and their earliest known fossil affiliates

A time-scaled phylogenetic analysis using only DNA sequence data requires the selection of justified fossil calibrators and the compilation of their lineage affiliations and probable ages (*e.g*., Table S1). Future studies of this type that elect to include sequences from all five genetically-characterized sea cow species (living and Recent) can calibrate three sirenian lineage splits. In the following, we filter our results to include only the earliest known fossil affiliates of the sirenian lineages that can be detected from this five-species genetic sample. The provided ages in this summary should be treated as minimum bounds where they are applicable to nodal diversifications.

(1) Our results identified the CBI-1-542 petrosal specimen from Chambi, Tunisia (Benoit et al., 2013) as the least derived and earliest known stem sirenian. The TE analysis yielded a median age estimate of 47.04 Ma for this unnamed taxon. *Prorastomus sirenoides* from Jamaica, here assessed as ~2.25 million years younger than the Chambi specimen, was formerly designated the oldest stem sea cow. (2) Given the TE results, the Puerto Rican occurrence of *Priscosiren atlantica* (Vélez-Juarbe & Domning, 2014b) represents the earliest known stem dugongid and is also the earliest known crown sirenian. The median age estimate for this occurrence was 29.45 Ma. Recent parsimony studies have also nested *Priscosiren* within crown Sirenia (*e.g*., Springer et al., 2015; Vélez-Juarbe & Wood, 2018). (3) Our analyses indicate that miosirenines from the late Oligocene and early Miocene of Europe are more likely to be stem sirenians than stem trichechids. Therefore, we consider the CTA-63/Pebas-base molar of an indisputable trichechid (Antoine et al., 2016) from near Contamana, Peru to be the earliest known stem trichechid. The age of the specimen is geologically constrained to 21-20.1 Ma with a median of 20.55 Ma (Dunn et al., 2013). (4) The indeterminate subspecies of *Trichechus manatus* (Domning, 2005) from the Leisley Shell Pits of Florida is the earliest known stem *T. manatus*. The geologic age of this taxon is tightly constrained with a likely range of 1.3-1.1 Ma and a median of 1.2 Ma. Given the DNA-inferred sister relationship of the extant manatee species in the Western Hemisphere (de Souza et al., 2021; this study), this specimen also informs a minimum age for the *T. manatus*-*T. inunguis* split. (5) In this study we defined crown Dugongidae by the *Dugong dugon*-*Hydrodamalis gigas* split. Further, we considered all species that were placed on the *Dugong* side of this diversification to be dugongines and all placed on the *Hydrodamalis* side to be hydrodamalines (Fig. 4). Our TE results identified *Metaxytherium crataegense* (Aranda-Manteca, Domning & Barnes, 1994) as the earliest known dugongine and also the earliest known crown dugongid. The oldest occurrence of this species is from Maryland with a median age estimate of 15.86 Ma. (6) Finally, *Dusisiren reinharti* (Domning, 1978) from Baja California Sur is the earliest known hydrodamaline with a median age estimate of 14.98 Ma.

## Conclusion

The phylogenetic and biogeographic models presented here suggest a new narrative for sea cow evolution throughout the Cenozoic. These results imply that the origin of Pan-Sirenia was in the Tethys during the latest Paleocene and that the evolution of the order’s earliest lineages was centered in the same region until the end of the Eocene, with only one surviving into the Oligocene. A late Ypresian or early Lutetian trans-Atlantic dispersal from the Tethys towards the Caribbean led to the Jamaican prorastomids, though it appears that Prorastomidae evolved in the Tethys prior to this event. The age of this dispersal implies that sirenians were the first marine mammals of the Western Hemisphere. We speculate that a second dispersal from the Tethys during the Bartonian led to at least some fossil occurrences of middle-to-late Eocene sea cows from eastern North America, though our dataset did not sample the material necessary to model this biogeographic transition. There is no indication that either of these dispersals left descendant lineages in the Western Hemisphere that survived beyond the Eocene.

During the late Eocene and from a Tethys origin, some sea cows moved into more northern latitudes of Europe. Our analyses suggest that this region became the point of departure for a terminal Eocene trans-Atlantic dispersal towards the Caribbean and northern-adjacent west Atlantic that led directly to the origin of crown Sirenia. The EOB age of this event correlates with the onset of severe climatic and eustatic events, and is coincident with widespread extinctions of both marine and terrestrial faunas. Some of the aforesaid European lineages survived the EOB, but with only one exception stem sirenians of the Eastern Hemisphere were extirpated before the close of the Oligocene.

The EOB founding of crown Sirenia in the Western Hemisphere gave rise to the Trichechidae and Dugongidae lineages. The earliest known fossil trichechid is from the early Miocene of Peru which indicates a ghost lineage spanning the entire Oligocene and the earliest portion of the Miocene. Our results suggest that undetected trichechids were probably present in the Caribbean Basin during the Oligocene, perhaps along the northern coast of South America. By the early Miocene, trichechids had moved into western Amazonia, presumably by way of a drainage connection between the Pebas mega-wetlands and the Caribbean (*i.e*., through Colombia and Venezuela). Our analyses suggest that from at least the early Miocene through the Pliocene trichechid evolution occurred entirely within South America’s mega-wetland systems. We infer that the Pliocene establishment of Amazon drainage into the South Atlantic was the event that permitted crown (or near-crown) trichechids to move out of the continent. Given our time-tree results, the trans-Atlantic dispersal of the *Trichechus senegalensis* lineage from South America to western Africa occurred sometime after the mid-Pliocene. The earliest spread of the *Trichechus manatus* lineage into the Caribbean and northern-adjacent west Atlantic probably occurred in the Pleistocene which highlights manatees as relatively recent additions to the aquatic communities of these regions. Further, our analyses indicate that by the time *T. manatus* came to inhabit the Caribbean, there were no remaining dugongids in the west Atlantic.

Soon after the crown Sirenia split, *in situ* dugongid diversity increased rapidly and reached a peak within the first five million years of the Oligocene. From the early Oligocene to the earliest Pliocene, several dugongid lineages simultaneously inhabited this region (*i.e*., the Caribbean and northern-adjacent west Atlantic). While not all of these taxa were necessarily sympatric, variable ecomorphological traits suggest that co-occurrences might be explained by differential foraging preferences. From this source region, our analyses detected two dugongid trans-Atlantic dispersals to the Eastern Hemisphere (mid-Oligocene and probably earliest Miocene), the earlier of which arrived in the Tethys and eventually led to a subsequent dispersal into the Indian Ocean—establishing the South Asian rytiodontine group. From the same west Atlantic source region, our results suggested two early Miocene dugongid dispersals into the Pacific, one of which gave rise to Hydrodamalinae. The diversity of hydrodamalines would peak near the end of the early Miocene and this clade would persist in the Pacific until the extinction of Steller’s sea cow in the mid-1700s. Our model suggests that the Indo-Pacific distribution of modern dugongs is the result of a trans-Pacific dispersal that originated near Florida and occurred sometime after the end of the middle Miocene. All available evidence indicates that west Atlantic dugongids disappeared before the end of the Pliocene.

While overall sirenian diversity seems to have peaked during the late Oligocene and early Miocene, the underlying geographic pattern provides a more informative characterization. These assessments found: (1) a diversity surge of dugongids in the Caribbean and northern-adjacent west Atlantic during the early Oligocene that led to a regional persistence of multispecies assemblages that lasted until the earliest Pliocene; (2) a rapid increase in hydrodamaline diversity after the group became established in the Pacific during the early Miocene; and (3) diminishing numbers of Afro-Arabian and European lineages from the EOB onward. Notably, sirenian diversity has declined by a minimum of 63% over the past nine million years.

From a methodological perspective, this study has demonstrated that analyses in which divergence times are estimated solely from molecular sequences and fossil-calibrated nodes can yield skewed results. Combining data types integrates morphological distances into the temporal model, thereby providing rate information about apomorphy acquisition, which improves upon the minimum-node-age approach. Fundamental to calculating morphological distances is the assignment of geologic ages (or age ranges) for all included taxa. Further, our results have illustrated that assessments of morphological data to the exclusion of DNA can yield topological and temporal estimates that are incompatible with rates of molecular evolution. At present, the only available phylogenetic method that accommodates all of the above is total evidence Bayesian tip-dating.

## Supplemental Information

10.7717/peerj.13886/supp-1 Supplemental Information 1 Consensus tree (allcompat) from the time-scaled Bayesian tip-dating analysis of the morphology [+ biogeographic character] supermatrix.

10.7717/peerj.13886/supp-2 Supplemental Information 2 Fossil calibrators for the time-scaled Bayesian phylogenetic analysis of the DNA supermatrix.

10.7717/peerj.13886/supp-3 Supplemental Information 3 Temporal and geospatial data, longitudinal zone codes, and posterior age ranges.

## Additional Information and Declarations

### Data Availability

The following information was supplied regarding data availability: All data, analysis settings, and additional supplemental files are available at the Open Science Framework: Heritage, Steven, and Erik R Seiffert. 2022. “Heritage S, Seiffert ER. (2022) OSF Data Supplement.” OSF. July 14. DOI 10.17605/OSF.IO/H7ZFD.
