# Supplementary material for: Total evidence time-scaled phylogenetic and biogeographic models for the evolution of sea cows (Sirenia, Afrotheria)
Source: PeerJ. 2022 Aug 25;10:e13886. doi: 10.7717/peerj.13886 (PMC9420408; doi:10.7717/peerj.13886)
Supplement: Supplemental Information 1 [file peerj-10-13886-s001.pdf]

Heritage & Seiffert (2022)

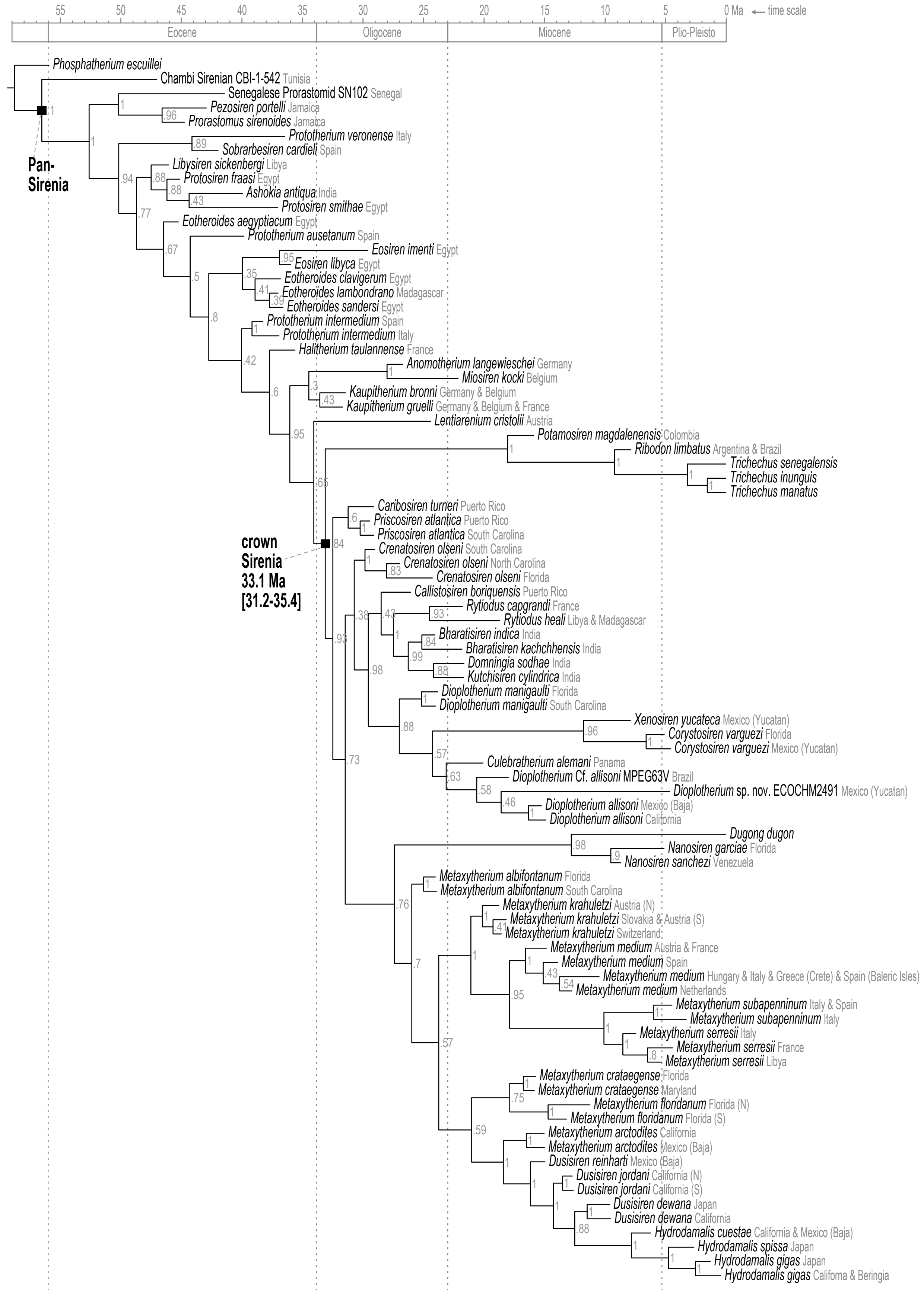

Figure S1. Consensus tree (allcompat) from the time-scaled Bayesian tip-dating analysis of the morphology [+ biogeographic character] supermatrix.
