# Supplementary material for: Total evidence time-scaled phylogenetic and biogeographic models for the evolution of sea cows (Sirenia, Afrotheria)
Source: PeerJ. 2022 Aug 25;10:e13886. doi: 10.7717/peerj.13886 (PMC9420408; doi:10.7717/peerj.13886)
Supplement: Supplemental Information 2 [file peerj-10-13886-s002.pdf]

**Table S1. Fossil calibrators for the time-scaled Bayesian phylogenetic analysis of the DNA supermatrix.**

| Fossil Taxon | Phylogenetic Placement | Site | Geologic Age |
|---|---|---|---|
| *Myorycteropus africanus* | oldest stem-Tubulidentata | Locality IV, Napak, Uganda | 19.5 [19-20] |
| *Todralestes variabilis* | oldest stem-Afrosoricida | Adar Mgorn 1, Ouarzazate, Morocco | 56.8 [56-57.6] |
| *Eochrysochloris tribosphenus* | oldest stem-Chrysochloridea | Quarry E, Jebel Qatrani Fm., Fayum, Egypt | 32.1 [31-33.2] |
| *Chambius kasserinensis* | oldest stem-Macroscelidea | CBI-1, Djebel Chambi, Kasserine Plateau, Tunisia | 46.8 [45.5-48.07] |
| *Metoldobotes* sp. | advanced stem-Macroscelidea | L-41, Jebel Qatrani Fm., Fayum, Egypt | 34.5 [33.9-35] |
| *Oligorhynchocyon songwensis* | oldest stem-Rhynchocyoninae | TZ-01S, Songwe Mbr., Nsungwe Fm., Tanzania | 25 [25-25] |
| *Seggeurius amourensis* | oldest stem-Hyracoidea | El Kohol, Algeria | 52.9 [51.3-54.5] |
| *Heterohyrax auricampensis* | earliest *Heterohyrax* | Site I, Berk Aukas, Namibia | 10.4 [9.7-11.1] |
| *Dendrohyrax samueli* | earliest *Dendrohyrax* | Aragai, Lukeino Fm., Tugen Hills, Kenya | 6 [6-6] |
| *Loxodonta* sp. indet. | earliest *Loxodonta* | Site 266, Toros-Menalla, Chad | 6.5 [6-7] |
| *Mammuthus subplanifrons* | earliest *Mammuthus* | Kuseralee Mbr., Sagantole Fm., Middle Awash, Ethiopia | 5.5 [5.2-5.8] |
| *Losodokodon losodokius* | oldest stem-Mammutidae | Eragaleit Beds, Lothidok, Kenya | 25.8 [24-27.5] |
| *Phosphatherium escuilliei* [a] | oldest stem-Proboscidea | Intercalary bed II/I, Sidi Chennane, Ouled Abdoun, Morocco | 56 [56-56] |
| *Priscosiren atlantica* [b] | oldest stem-Dugongidae | LACM 8060, Rio Guatemala Sect., San Sebastian Fm., Puerto Rico | 29.5 [29.17-29.78] |
| Chambi Sirenian (CBI-1-542) | oldest stem-Sirenia | CBI-1, Djebel Chambi, Kasserine Plateau, Tunisia | 46.8 [45.5-48.07] |
| *Eritherium azzouzorum* | oldest crown-Afrotheria | Bed IIa, Sidi Chennane, Ouled Abdoun, Morocco | 60 [60-60] |

**Notes:**

[a] alternate for *Daouitherium rebouli*, same site & age
[b] occurrence in Puerto Rico
Ages reported in millions of years
