# Supplementary material for: Total evidence time-scaled phylogenetic and biogeographic models for the evolution of sea cows (Sirenia, Afrotheria)
Source: PeerJ. 2022 Aug 25;10:e13886. doi: 10.7717/peerj.13886 (PMC9420408; doi:10.7717/peerj.13886)
Supplement: Supplemental Information 3 [file peerj-10-13886-s003.pdf]

**Table S2. Temporal and Geospatial Data, Longitudinal Zone Codes, and Posterior Age Ranges.**

| Map ID | Fossil Taxon | Location | Longitude | Latitude | Zone | Geologic Age Min | Geologic Age Max | 68 HDI Age [a] Lower | 68 HDI Age [a] Upper | 68 HDI Age [b] Lower | 68 HDI Age [b] Upper |
|---|---|---|---|---|---|---|---|---|---|---|---|
| 1 | *Anomotherium langewieschei* | Germany | 9.4961 | 51.3189 | 3 | 25.36 | 28.94 | 25.360 | 27.342 | 25.360 | 27.359 |
| 2 | *Ashokia antiqua* | India | 68.7500 | 23.4806 | 4 | 39 | 41.03 | 39.446 | 40.816 | 39.003 | 40.363 |
| 3 | *Bharatisiren indica* | India | 68.9500 | 23.5436 | 4 | 23.55 | 26.9 | 23.550 | 24.422 | 23.550 | 24.352 |
| 4 | *Bharatisiren kachchhensis* | India | 68.8000 | 23.4028 | 4 | 20.45 | 23.04 | 21.384 | 23.040 | 21.365 | 23.040 |
| 5 | *Callistosiren boriquensis* | Puerto Rico | -66.9465 | 18.3329 | 2 | 24.7 | 27.17 | 25.732 | 27.170 | 25.645 | 27.170 |
| 6 | *Caribosiren turneri* | Puerto Rico | -66.9167 | 18.2833 | 2 | 27.17 | 30.51 | 28.499 | 30.505 | 28.575 | 30.509 |
| 7 | Chambi Sirenian CBI-1-542 | Tunisia | 8.6547 | 35.2119 | 3 | 45.4 | 48.07 | 46.551 | 48.070 | 46.585 | 48.070 |
| 8 | *Corystosiren varguezi* | Florida | -81.8000 | 27.9000 | 2 | 4.9 | 5.3 | 4.901 | 5.170 | 4.901 | 5.171 |
| 9 | *Corystosiren varguezi* | Mexico (Yucatan) | -87.8167 | 21.1833 | 2 | 3.5 | 5.33 | 4.210 | 5.330 | 4.256 | 5.330 |
| 10 | *Crenatosiren olseni* | Florida | -82.7900 | 30.3300 | 2 | 23.4 | 24.9 | 23.983 | 24.900 | 24.002 | 24.900 |
| 11 | *Crenatosiren olseni* | North Carolina | -77.2686 | 34.5703 | 2 | 26.5 | 27.5 | 26.504 | 27.168 | 26.500 | 27.165 |
| 12 | *Crenatosiren olseni* | South Carolina | -80.1517 | 32.9672 | 2 | 28.75 | 29.5 | 28.750 | 29.201 | 28.750 | 29.185 |
| 13 | *Culebratherium alemani* | Panama | -79.6600 | 9.0500 | 2 | 19.12 | 21.24 | 19.120 | 20.470 | 19.120 | 20.470 |
| 14 | *Dioplotherium allisoni* | California | -117.7522 | 33.5575 | 1 | 13.82 | 15.99 | 13.963 | 15.390 | 14.143 | 15.568 |
| 15 | *Dioplotherium allisoni* | Mexico (Baja) | -112.0000 | 26.3500 | 1 | 13.82 | 18.22 | 13.821 | 15.943 | 13.829 | 15.833 |
| 16 | *Dioplotherium* Cf. *D. allisoni* MPEG 63-V | Brazil | -47.2875 | -0.5958 | 2 | 15.99 | 20.45 | 15.991 | 18.801 | 15.990 | 18.771 |
| 17 | *Dioplotherium manigaulti* | Florida | -82.2600 | 29.3900 | 2 | 23.07 | 25.17 | 23.070 | 24.247 | 23.070 | 24.173 |
| 18 | *Dioplotherium manigaulti* | South Carolina | -80.1069 | 32.8775 | 2 | 23.07 | 25.17 | 23.070 | 24.368 | 23.070 | 24.370 |
| 19 | *Dioplotherium* sp. nov. ECOCHM-2491 | Mexico (Yucatan) | -87.8167 | 21.1833 | 2 | 3.5 | 5.33 | 4.285 | 5.330 | 4.329 | 5.330 |
| 20 | *Domningia sodhae* | India | 68.9144 | 23.3722 | 4 | 20.45 | 23.04 | 20.507 | 22.189 | 20.451 | 22.122 |
| 21 | *Dusisiren dewana* | California | -122.7250 | 37.9033 | 1 | 9 | 10 | 9.348 | 9.999 | 9.361 | 10.000 |
| 22 | *Dusisiren dewana* | Japan | 140.2111 | 38.3317 | 0 | 9 | 10.4 | 9.001 | 9.902 | 9.000 | 9.884 |
| 23 | *Dusisiren jordani* | California | -122.0505 | 37.0715 | 1 | 11.95 | 13.6 | 12.063 | 13.098 | 12.017 | 13.030 |
| 24 | *Dusisiren jordani* | California | -117.7122 | 33.5619 | 1 | 11.95 | 13.6 | 11.950 | 12.903 | 11.950 | 12.888 |
| 25 | *Dusisiren reinharti* | Mexico (Baja) | -112.5000 | 26.2333 | 1 | 13.82 | 15.99 | 14.551 | 15.932 | 14.547 | 15.938 |
| 26 | *Eosiren imenti* | Egypt | 30.7004 | 29.6435 | 3 | 29.18 | 29.97 | 29.471 | 29.970 | 29.475 | 29.970 |
| 27 | *Eosiren libyca* | Egypt | 30.7004 | 29.6219 | 3 | 35.1 | 37.39 | 35.100 | 36.426 | 35.100 | 36.411 |
| 28 | *Eotheroides aegyptiacum* | Egypt | 31.2720 | 30.3600 | 3 | 44.7 | 46 | 44.700 | 45.552 | 44.700 | 45.529 |
| 29 | *Eotheroides clavigerum* | Egypt | 30.0566 | 29.3036 | 3 | 35.34 | 38.08 | 36.367 | 38.080 | 36.348 | 38.080 |
| 30 | *Eotheroides lambondrano* | Madagascar | 46.4206 | -15.6339 | 3 | 33.9 | 48.07 | 33.903 | 38.858 | 33.900 | 38.438 |
| 31 | *Eotheroides sandersi* | Egypt | 30.0249 | 29.2709 | 3 | 35.34 | 38.08 | 35.351 | 37.120 | 35.423 | 37.195 |
| 32 | *Halitherium taulannense* | France | 6.4591 | 43.8765 | 3 | 33.9 | 36.5 | 35.146 | 36.500 | 35.197 | 36.500 |
| 33 | *Hydrodamalis cuestae* | California | -120.7333 | 35.1667 | 1 | 3.6 | 7.25 | 5.529 | 7.250 | 5.660 | 7.250 |
| 34 | *Hydrodamalis cuestae* | California | -117.7047 | 33.6353 | 1 | 3.6 | 7.25 | 5.529 | 7.250 | 5.660 | 7.250 |
| 35 | *Hydrodamalis cuestae* | Mexico (Baja) | -115.7333 | 30.0667 | 1 | 3.6 | 7.25 | 5.529 | 7.250 | 5.660 | 7.250 |
| 36 | *Hydrodamalis gigas* | Aleutian Islands | -176.6403 | 51.7844 | 0 | 0.00025 | 0.78 | 0.256 | 0.780 | 0.278 | 0.780 |
| 37 | *Hydrodamalis gigas* | Aleutian Islands | 178.9833 | 51.5422 | 0 | 0.00025 | 0.78 | 0.256 | 0.780 | 0.278 | 0.780 |
| 38 | *Hydrodamalis gigas* | Aleutian Islands | 172.9306 | 52.8922 | 0 | 0.00025 | 0.78 | 0.256 | 0.780 | 0.278 | 0.780 |
| 39 | *Hydrodamalis gigas* | Aleutian Islands | 175.9250 | 52.3556 | 0 | 0.00025 | 0.78 | 0.256 | 0.780 | 0.278 | 0.780 |
| 40 | *Hydrodamalis gigas* | Aleutian Islands | 177.4564 | 51.9689 | 0 | 0.00025 | 0.78 | 0.256 | 0.780 | 0.278 | 0.780 |
| 41 | *Hydrodamalis gigas* | Aleutian Islands | -171.7000 | 63.7000 | 0 | 0.00025 | 0.78 | 0.256 | 0.780 | 0.278 | 0.780 |
| 42 | *Hydrodamalis gigas* | California | -121.9900 | 36.9325 | 1 | 0.00025 | 0.78 | 0.256 | 0.780 | 0.278 | 0.780 |
| 43 | *Hydrodamalis gigas* | Japan | 141.5374 | 42.8056 | 0 | 0.78 | 1.8 | 1.127 | 1.800 | 1.100 | 1.789 |
| 44 | *Hydrodamalis gigas* | Japan | 140.1478 | 35.3439 | 0 | 0.78 | 1.8 | 1.127 | 1.800 | 1.100 | 1.789 |
| 45 | *Hydrodamalis gigas* | Bering Island | 166.2500 | 55.0000 | 0 | 0.00025 | 0.78 | 0.256 | 0.780 | 0.278 | 0.780 |
| 46 | *Hydrodamalis spissa* | Japan | 144.0897 | 43.1789 | 0 | 1.8 | 5.33 | 1.800 | 3.292 | 1.800 | 3.107 |
| 47 | *Kaupitherium bronni* | Belgium | 4.4000 | 51.1000 | 3 | 27.29 | 33.9 | 29.674 | 33.468 | 30.489 | 33.889 |
| 48 | *Kaupitherium bronni* | Germany | 8.3000 | 50.0000 | 3 | 27.29 | 33.9 | 29.674 | 33.468 | 30.489 | 33.889 |
| 49 | *Kaupitherium bronni* | Germany | 12.5000 | 51.2000 | 3 | 27.29 | 33.9 | 29.674 | 33.468 | 30.489 | 33.889 |
| 50 | *Kaupitherium bronni* | Germany | 6.9000 | 51.6000 | 3 | 27.29 | 33.9 | 29.674 | 33.468 | 30.489 | 33.889 |
| 51 | *Kaupitherium gruelli* | Belgium | 4.4000 | 51.1000 | 3 | 27.29 | 33.9 | 30.166 | 33.900 | 30.702 | 33.900 |
| 52 | *Kaupitherium gruelli* | France | 1.6000 | 48.6000 | 3 | 27.29 | 33.9 | 30.166 | 33.900 | 30.702 | 33.900 |
| 53 | *Kaupitherium gruelli* | Germany | 8.3000 | 50.0000 | 3 | 27.29 | 33.9 | 30.166 | 33.900 | 30.702 | 33.900 |

**Notes:**

**continued…**

[a] 68% posterior highest density interval from analysis of the total evidence supermatrix

[b] 68% posterior highest density interval from analysis of the morphology [+ biogeographic character] supermatrix

Ages reported in millions of years

**Table S2.** (continued)

| Map ID | Fossil Taxon | Location | Longitude | Latitude | Zone | Geologic Age Min | Max | 68 HDI Age [a] Lower | Upper | 68 HDI Age [b] Lower | Upper |
|---|---|---|---|---|---|---|---|---|---|---|---|
| 54 | *Kaupitherium gruelli* | Germany | 6.9000 | 51.6000 | 3 | 27.29 | 33.9 | 30.166 | 33.900 | 30.702 | 33.900 |
| 55 | *Kutchisiren cylindrica* | India | 68.9125 | 23.4833 | 4 | 20.45 | 23.04 | 20.793 | 22.499 | 20.613 | 22.312 |
| 56 | *Lentiarenium cristolii* | Austria | 14.2861 | 48.3061 | 3 | 23.04 | 25.17 | 23.991 | 25.170 | 24.047 | 25.170 |
| 57 | *Libysiren sickenbergi* | Libya | 18.5000 | 28.2500 | 3 | 45.4 | 48.07 | 45.400 | 46.559 | 45.400 | 46.449 |
| 58 | *Metaxytherium albifontanum* | Florida | -82.7350 | 30.3290 | 2 | 23.07 | 25.17 | 23.343 | 24.685 | 23.172 | 24.477 |
| 59 | *Metaxytherium albifontanum* | South Carolina | -80.0575 | 33.0082 | 2 | 23.07 | 25.17 | 23.071 | 24.353 | 23.071 | 24.286 |
| 60 | *Metaxytherium arctodites* | California | -117.6244 | 33.6933 | 1 | 13.6 | 16.3 | 14.206 | 15.949 | 14.357 | 16.066 |
| 61 | *Metaxytherium arctodites* | Mexico (Baja) | -116.8000 | 32.1167 | 1 | 13.6 | 16.3 | 14.002 | 15.750 | 14.365 | 16.089 |
| 62 | *Metaxytherium crataegense* | Florida | -84.6000 | 30.6000 | 2 | 15 | 16.3 | 15.493 | 16.297 | 15.507 | 16.300 |
| 63 | *Metaxytherium crataegense* | Maryland | -76.5200 | 38.6000 | 2 | 15.5 | 16.25 | 15.500 | 15.991 | 15.500 | 15.988 |
| 64 | *Metaxytherium floridanum* | Florida | -82.3900 | 29.6600 | 2 | 9.3 | 11.95 | 10.796 | 11.950 | 10.853 | 11.950 |
| 65 | *Metaxytherium floridanum* | Florida | -82.1300 | 27.6700 | 2 | 11.95 | 14.6 | 11.950 | 13.585 | 11.950 | 13.659 |
| 66 | *Metaxytherium krahuletzi* | Austria | 15.8177 | 48.6397 | 3 | 18.2 | 19.8 | 17.790 | 18.949 | 17.863 | 19.000 |
| 67 | *Metaxytherium krahuletzi* | Austria | 15.6720 | 48.7979 | 3 | 17.2 | 19 | 18.200 | 19.094 | 18.200 | 19.050 |
| 68 | *Metaxytherium krahuletzi* | Slovakia | 19.3327 | 48.2780 | 3 | 17.2 | 19 | 17.790 | 18.949 | 17.863 | 19.000 |
| 69 | *Metaxytherium krahuletzi* | Switzerland | 8.2286 | 47.4092 | 3 | 17.7 | 19.8 | 17.700 | 18.921 | 17.700 | 18.909 |
| 70 | *Metaxytherium medium* | Austria | 15.6000 | 46.7000 | 3 | 13.82 | 16 | 13.965 | 15.421 | 13.820 | 15.228 |
| 71 | *Metaxytherium medium* | France | -0.0560 | 47.5380 | 3 | 13.82 | 16 | 13.965 | 15.421 | 13.820 | 15.228 |
| 72 | *Metaxytherium medium* | Greece (Crete) | 26.2000 | 35.2000 | 3 | 7.7 | 11.65 | 9.660 | 11.650 | 9.838 | 11.650 |
| 73 | *Metaxytherium medium* | Hungary | 19.7000 | 48.0000 | 3 | 7.7 | 11.65 | 9.660 | 11.650 | 9.838 | 11.650 |
| 74 | *Metaxytherium medium* | Italy | 10.9000 | 43.4000 | 3 | 7.7 | 11.1 | 9.660 | 11.650 | 9.838 | 11.650 |
| 75 | *Metaxytherium medium* | Italy (Calabria) | 15.9200 | 38.6700 | 3 | 7.7 | 11.65 | 9.660 | 11.650 | 9.838 | 11.650 |
| 76 | *Metaxytherium medium* | Netherlands | 6.6000 | 52.1000 | 3 | 11.65 | 13.82 | 11.867 | 13.325 | 11.868 | 13.333 |
| 77 | *Metaxytherium medium* | Spain (Balearic Isles) | 3.1000 | 39.7000 | 3 | 7.7 | 11.65 | 9.660 | 11.650 | 9.838 | 11.650 |
| 78 | *Metaxytherium medium* | Spain | 1.7000 | 41.3000 | 3 | 12.5 | 16 | 12.500 | 14.488 | 12.500 | 14.498 |
| 79 | *Metaxytherium serresii* | France | 3.8792 | 43.6086 | 3 | 4 | 4.75 | 4.264 | 4.750 | 4.269 | 4.750 |
| 80 | *Metaxytherium serresii* | Italy | 15.9306 | 38.6658 | 3 | 7.3 | 7.6 | 7.301 | 7.503 | 7.300 | 7.502 |
| 81 | *Metaxytherium serresii* | Libya | 20.7494 | 30.1392 | 3 | 4.83 | 5.83 | 4.830 | 5.503 | 4.836 | 5.513 |
| 82 | *Metaxytherium subapenninum* | Italy (Tuscany) | 11.2519 | 42.8006 | 3 | 3.6 | 5.33 | 4.116 | 5.285 | 3.601 | 4.763 |
| 83 | *Metaxytherium subapenninum* | Italy | 7.8544 | 44.6978 | 3 | 2.58 | 3.8 | 3.024 | 3.800 | 3.053 | 3.800 |
| 84 | *Metaxytherium subapenninum* | Spain | -1.3136 | 37.5992 | 3 | 3.6 | 5.33 | 4.116 | 5.285 | 3.601 | 4.763 |
| 85 | *Miosiren kocki* | Belgium | 4.3636 | 51.0861 | 3 | 21.12 | 22.94 | 21.816 | 22.940 | 21.813 | 22.940 |
| 86 | *Nanosiren garciae* | Florida | -82.1100 | 27.6200 | 2 | 4.9 | 5.3 | 5.039 | 5.300 | 5.041 | 5.300 |
| 87 | *Nanosiren sanchezi* | Venezuela | -70.2772 | 11.2492 | 2 | 8 | 10 | 8.000 | 8.918 | 8.000 | 9.050 |
| 88 | *Pezosiren portelli* | Jamaica | -77.9167 | 18.3333 | 2 | 41.95 | 44 | 42.621 | 43.990 | 42.323 | 43.702 |
| 89 | *Potamosiren magdalenensis* | Colombia | -75.1667 | 3.3333 | 2 | 15.5 | 16.3 | 15.734 | 16.275 | 15.759 | 16.300 |
| 90 | *Priscosiren atlantica* | Puerto Rico | -67.0050 | 18.3383 | 2 | 29.17 | 29.78 | 29.171 | 29.560 | 29.170 | 29.557 |
| 91 | *Priscosiren atlantica* | South Carolina | -80.0575 | 33.0082 | 2 | 28.75 | 29.5 | 29.002 | 29.500 | 29.010 | 29.500 |
| 92 | *Prorastomus sirenoides* | Jamaica | -77.5000 | 18.1833 | 2 | 43.65 | 46.25 | 43.650 | 45.225 | 43.650 | 45.254 |
| 93 | *Protosiren fraasi* | Egypt | 31.2720 | 30.3600 | 3 | 44.7 | 46 | 44.700 | 45.415 | 44.700 | 45.390 |
| 94 | *Protosiren smithae* | Egypt | 30.0570 | 29.3030 | 3 | 35.34 | 38.08 | 36.574 | 38.080 | 36.580 | 38.080 |
| 95 | *Prototherium ausetanum* | Spain | 2.2159 | 41.8413 | 3 | 37.41 | 41.03 | 39.180 | 41.030 | 39.242 | 41.030 |
| 96 | *Prototherium intermedium* | Italy | 11.8667 | 45.8500 | 3 | 35.81 | 37.71 | 36.584 | 37.710 | 36.611 | 37.710 |
| 97 | *Prototherium intermedium* | Spain | 2.2333 | 41.8500 | 3 | 37.71 | 39.37 | 37.710 | 38.625 | 37.710 | 38.596 |
| 98 | *Prototherium veronense* | Italy | 11.3742 | 45.6339 | 3 | 33.9 | 37.71 | 35.783 | 37.710 | 35.823 | 37.710 |
| 99 | *Ribodon limbatus* | Argentina | -59.2500 | -33.7167 | 2 | 6.8 | 9 | 6.802 | 8.251 | 6.800 | 8.248 |
| 100 | *Ribodon limbatus* | Brazil | -66.8794 | -10.1164 | 2 | 6.8 | 9 | 6.802 | 8.251 | 6.800 | 8.248 |
| 101 | *Rytiodus capgrandi* | France | 0.4608 | 44.3681 | 3 | 20.45 | 23.04 | 21.342 | 23.040 | 21.332 | 23.040 |
| 102 | *Rytiodus heali* | Libya | 19.7300 | 28.7600 | 3 | 13.82 | 20.45 | 17.679 | 20.450 | 17.738 | 20.450 |
| 103 | *Rytiodus heali* | Madagascar | 45.9026 | -15.7153 | 3 | 13.82 | 20.45 | 17.679 | 20.450 | 17.738 | 20.450 |
| 104 | Senegalese Prorastomid SN102 | Senegal | -16.8749 | 15.0437 | 3 | 41.03 | 41.85 | 41.317 | 41.850 | 41.323 | 41.850 |
| 105 | *Sobrarbesiren cardieli* | Spain | 0.1305 | 42.3234 | 3 | 41.8 | 42.2 | 41.815 | 42.085 | 41.808 | 42.080 |
| 106 | *Xenosiren yucateca* | Mexico (Yucatan) | -89.7167 | 21.0667 | 2 | 4.9 | 10.3 | 6.538 | 10.095 | 6.938 | 10.300 |

**Notes:**

[a] 68% posterior highest density interval from analysis of the total evidence supermatrix
[b] 68% posterior highest density interval from analysis of the morphology [+ biogeographic character] supermatrix
Ages reported in millions of years
